# Are intersectoral costs considered in economic evaluations of interventions relating to sexually transmitted infections (STIs)? A systematic review

**DOI:** 10.1186/s12889-022-14484-z

**Published:** 2022-11-25

**Authors:** Lena Schnitzler, Silvia M. A. A. Evers, Louise J. Jackson, Aggie T. G. Paulus, Tracy E. Roberts

**Affiliations:** 1 Health Economics Unit, Institute of Applied Health Research, College of Medical and Dental Sciences, University of Birmingham, Birmingham, UK; 2 Department of Health Services Research, Care and Public Health Research Institute (CAPHRI), Faculty of Health, Medicine and Life Sciences (FHML), Maastricht University, Maastricht, The Netherlands; 3 Trimbos Institute, Netherlands Institute of Mental Health and Addiction, Utrecht, The Netherlands; 4 School of Health Professions Education (SHE), Faculty of Health, Medicine and Life Sciences (FHML), Maastricht University, Maastricht, The Netherlands

**Keywords:** Intersectoral, Multisectoral, Societal, Costs, Health economics, HTA, Economic evaluation, Sexually transmitted infections, STIs, HIV

## Abstract

**Background/objective:**

Sexually transmitted infections (STIs) not only have an impact on the health sector but also the private resources of those affected, their families and other sectors of society (i.e. labour, education). This study aimed to i) review and identify economic evaluations of interventions relating to STIs, which aimed to include a societal perspective; ii) analyse the intersectoral costs (i.e. costs broader than healthcare) included; iii) categorise these costs by sector; and iv) assess the impact of intersectoral costs on the overall study results.

**Methods:**

Seven databases were searched: MEDLINE (PubMed), EMBASE (Ovid), Web of Science, CINAHL, PsycINFO, EconLit and NHS EED. Key search terms included terms for economic evaluation, STIs and specific infections. This review considered trial- and model-based economic evaluations conducted in an OECD member country. Studies were included that assessed intersectoral costs. Intersectoral costs were extracted and categorised by sector using Drummond’s cost classification scheme (i.e. patient/family, productivity, costs in other sectors). A narrative synthesis was performed.

**Results:**

Twenty-nine studies qualified for data extraction and narrative synthesis. Twenty-eight studies applied a societal perspective of which 8 additionally adopted a healthcare or payer perspective, or both. One study used a modified payer perspective. The following sectors were identified: patient/family, informal care, paid labour (productivity), non-paid opportunity costs, education, and consumption. Patient/family costs were captured in 11 studies and included patient time, travel expenses, out-of-pocket costs and premature burial costs. Informal caregiver support (non-family) and unpaid help by family/friends was captured in three studies. Paid labour losses were assessed in all but three studies. Three studies also captured the costs and inability to perform non-paid work. Educational costs and future non-health consumption costs were each captured in one study. The inclusion of intersectoral costs resulted in more favourable cost estimates.

**Conclusions:**

This systematic review suggests that economic evaluations of interventions relating to STIs that adopt a societal perspective tend to be limited in scope. There is an urgent need for economic evaluations to be more comprehensive in order to allow policy/decision-makers to make better-informed decisions.

**Supplementary Information:**

The online version contains supplementary material available at 10.1186/s12889-022-14484-z.

## Background

Sexually transmitted infections (STIs) continue to rise worldwide and generate important impacts on society [1]. STIs and their sequelae are shown to not only have an impact on the health sector but also the private resources of those affected, their families and other sectors of society [2]. Living with an STI can, for instance, affect an individual’s productivity and participation in the labour market [3–5]. It can also have a considerable impact on an individual’s mental health (i.e. stigma, depression), compromising an individual’s overall quality of life [6–8]. The wider societal impacts spilling over to other non-health sectors are also referred to as *societal* [9], *multisectoral* [10] or *intersectoral* costs and benefits (or consequences) [11, 12]. This review will use the term *intersectoral* costs.

Interventions relating to STIs are essential but complex in nature (like many other public health problems). This complexity can, in part, be explained by the aforementioned wide-ranging, intersectoral impacts of public health programmes, and this can create challenges in adequately capturing them in economic evaluations [12, 13]. The study perspective (or viewpoint) adopted in an economic evaluation ultimately determines the costs and benefits included in the analysis. A societal perspective is increasingly being advocated for economic evaluations of public health interventions as it is expected to capture all relevant costs and benefits associated with an intervention both within and beyond health [14, 15]. Depending on the study objective and stakeholder interests, it may be appropriate to assess costs and benefits from other perspectives (i.e. when estimating the financial costs of an intervention to a specific healthcare system or provider).

Even though a societal perspective has been advocated in methodological guidelines for some time [16], it is not always adopted or, if attempted, tends to be incomplete [17–21]. As indicated above, this can be due to the methodological challenges associated with quantifying the intersectoral impacts of public health interventions [22]. However, evidence suggests that the societal costs associated with STI-related interventions can substantially contribute to the total economic cost burden [2, 3]. This implies that excluding intersectoral costs from analyses could severely underestimate the total cost burden and present incomplete economic information to policy/decision-makers, potentially leading to suboptimal decision-making (i.e. inefficient allocation of resources) [18, 19, 23]. Hence, more comprehensive economic evidence is needed that will allow policy/decision-makers to better understand the total cost burden associated with STIs.

Given the importance placed on the societal perspective and the potential for suboptimal policy decisions in the absence of a comprehensive evaluation, a systematic review of published economic evaluations which adopted a societal perspective was undertaken. The review aimed to explore the intersectoral costs considered under a societal perspective in economic evaluations of interventions relating to STIs, and the impact of including intersectoral costs on the overall study results. The specific objectives of this review were to i) identify economic evaluations of interventions relating to STIs which aimed to include a societal perspective; ii) analyse the intersectoral costs (i.e. costs broader than healthcare) included; iii) categorise these costs by sector (i.e. patient/family, productivity, other); and iv) assess the impact of intersectoral costs on the overall study results.

## Methods

A protocol for this review was published in PROSPERO, the International Prospective Register of Systematic Reviews database (CRD42019130940). The review systematically followed the Centre for Review and Dissemination (CRD) guidance for undertaking reviews in health care [24] and a five-step approach on how to prepare a systematic review of economic evaluations for informing evidence-based healthcare decisions [25–27]. The PRISMA guidelines were followed for the reporting of this review [28] (supplemental file 1).

### Search strategy

A search strategy was developed in PubMed for MEDLINE together with an information specialist before adapting for use in other databases (supplemental file 2). Seven electronic databases were searched (1999–2019): MEDLINE (via PubMed), EMBASE (via Ovid), Web of Science (Core Collection), CINAHL, PsycINFO, EconLit and NHS EED. The search strategy was updated for 2020–21 in Medline (PubMed) only. The year 1999 was initially chosen as a starting year to reflect the inception of the United Kingdom (UK) National Institute of Health and Care Excellence (NICE) and their implementation of guidance statements for the conduct of health economic evaluations. Key search terms included terms for economic evaluation and STIs including specific infections.

### Inclusion criteria

This review considered both trial- and model-based economic evaluations of any intervention relating to STIs that were conducted in an Organisation for Economic Co-operation and Development (OECD) member country. OECD member countries were chosen due to their similarities in terms of health(care) systems and to better compare the methodology of studies concerned with similar health(care) systems. It focused on full economic evaluations that adopted a societal perspective, including cost-effectiveness analyses (CEA), cost-utility analyses (CUA) and cost-benefit analyses (CBA). The methods of CEA, CUA and CBA have varying theoretical foundations and outcomes are expressed differently. A CEA measures assesses outcomes (effects) in natural units, whereas a CUA assesses outcomes in health utilities such as quality-adjusted life years (QALYs). A CBA assesses both costs and outcomes in monetary values. No restrictions were placed on the type of comparator or outcomes. Participants in an intervention had to be at least 10 years of age to reflect international definitions of the start of adolescence (a period during which individuals establish sexual maturation and sexual activity) [29] (the PICO model is shown in supplemental file 3).

### Screening of data and data extraction

**Fig. 1:**
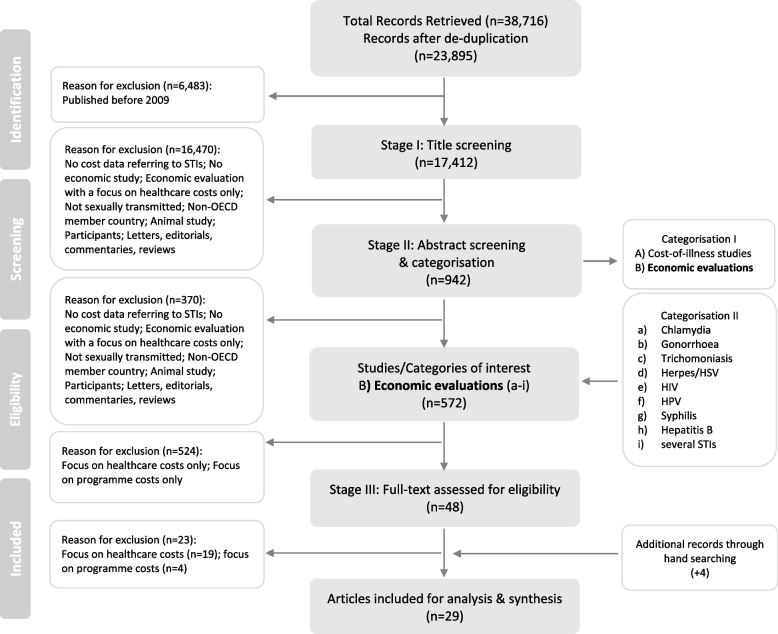
PRISMA Flowchart

Search results were exported into EndNote X9. Citations were de-duplicated following the guidelines by Bramer and colleagues [30]. The study selection was performed by two reviewers (LS, LJ). A systematic process was adopted to guide the screening of studies for inclusion [31]. Stage I: title screening (LS), stage II: abstract screening and categorisation of selected studies by study type and disease group (LS), and stage III: full-text screening (LS). This process was checked by a second reviewer (LJ) and discrepancies were discussed. A standardised data extraction sheet was utilised to record data on study characteristics, intersectoral costs and cost-effectiveness estimates for those studies that adopted a societal perspective in addition to a healthcare and/or provider perspective (to illustrate the difference in study results for each perspective) [27]. Corresponding authors of selected studies were contacted for clarification where it was not clear what types of costs were considered. A PRISMA flowchart illustrates the selection process (Fig. 1).

### Analysis

Data were recorded using Microsoft Excel and Word. Intersectoral costs were identified, extracted and categorised by sector using Drummond’s sector-specific cost classification scheme [16] (Table 1). Drummond and colleagues categorise costs into (i) healthcare, (ii) patient and family, (iii) productivity, and (iv) costs in other sectors such as informal care, educational costs, costs in the criminal justice system, household and/or leisure costs [11]. The reported intersectoral costs were converted to US Dollars and the year 2021, adjusting the values by inflation. This was done using an online inflation tool [32] and a currency converter [33]. A narrative synthesis was performed following CRD guidelines [24].

**Table 1:** Sector-specific cost classification scheme based on Drummond et al. [16]

Sector	Examples of cost components/resource items
Healthcare	i.e. treatment, medication, hospitalisation, other
Patient & family	i.e. patient time, out-of-pocket costs, travel expenses, other
Productivity	i.e. lost working days (labour costs), lost income, other
Costs in other sectors	i.e. education, criminal justice, leisure, household, informal care, other*

*Examples are based on Drost et al. [11] and Edwards et al. [12]

## Results

The search strategy generated 23,895 studies after duplicates were removed. Studies were further limited to those published from 2009 onwards, excluding 6483. Though this was due to a high number of records identified in the databases, the publication date reflects the period of time during which intersectoral costs and benefits have gained more prominence [13, 22, 34]. Titles and then abstracts were screened, and a total of 572 economic evaluations were identified. Studies were further screened to exclude evaluations that considered healthcare or intervention costs only. In total, 48 studies were identified for full text screening and 29 studies were taken forward for data extraction and narrative synthesis (see supplemental file 4 for excluded studies).

### Study characteristics

Of the 29 studies identified, the majority focused on HPV (*n* = 11), HIV (*n* = 8) and chlamydia (*n* = 7, of which two focused on both chlamydia and gonorrhoea) (Table 2). The remaining studies were concerned with gonorrhoea (*n* = 1), hepatitis B (*n* = 1) and hepatitis C (*n* = 1). The countries of interest in the selected studies included the United States of America (USA) (*n* = 10), The Netherlands (*n* = 8), Canada (*n* = 3), Sweden (*n* = 3), Germany (*n* = 2), Australia (*n* = 1), Austria (*n* = 1) and Israel (*n* = 1). The overwhelming approach adopted was a CEA (considering the study authors’ definition of their study as a CEA and CUA). However, not all studies explicitly stated whether they applied a CEA or CUA approach. Only three out of the 29 studies explicitly reported to have undertaken a CUA [35–37] and one study was explicit about having conducted both a CEA and CUA [38]. Modelling was used in all but two studies, which were trial-based [36, 37]. Most of the modelling studies applied a (dynamic) transmission model, which is the preferred method when evaluating infectious diseases [39–47]. Twenty-eight studies applied a societal perspective of which 8 additionally adopted a healthcare or payer perspective, or both [37, 39, 40, 44, 47–49]. One study used a modified payer perspective [50]. More information is presented in Table 2.

**Table 2:** Study characteristics

	Authors	Year	Country	Type of STI	Perspective	Type of intervention	Comparator	Type of analysis*	Type of study	Outcome(s)	Year of valuation	Currency
1	Adamson et al.	2019	USA	HIV	Multiple (Societal, Healthcare)	Financial incentives for HIV viral suppression	Standard of care	CEA	Disease progression model and ongoing transmission	QALYs, viral suppression, reduced HIV infections prevented	2017	USD
2	Campos et al.	2021	USA	HPV	Modified payer perspective	HPV testing self-collection at home	Standard of care involving cytology and HPV co-testing at the Health Department clinics	CEA	Monte Carlo microsimulation model/ micro-costing study alongside RCT	Year of life saved	2019	USD
3	Coupe et al.	2009	NL	HPV	Societal	Cervical cancer screening strategies	Vaccination only	Not explicitly stated	Markov simulation model	QALYs	2006	EUR
4	Damm et al.	2017	GER	HPV	Multiple (Societal, Healthcare)	Vaccination in addition to screening	Screening alone	Not explicitly stated	Dynamic transmission model (SIRS)	LYs, QALYs	2010	EUR
5	De Kok et al.	2009	NL	HPV	Societal	Vaccination in addition to cervical cancer screening	Screening alone	CEA	Simulation model (MISCAN)	QALYs, CIN lesions detected, cervical cancer diagnosis, cervical cancer deaths, life-years lost	2008	EUR
6	De Wit et al.	2015	NL	Chlamydia	Societal	Screening (six scenarios)	Another scenario, or no screening	Not explicitly stated	Analogous to the transmission dynamics model	QALYs	2010	EUR
7	Deogan et al.	2010	SWE	Chlamydia	Societal	Community based intervention (testing, treatment, contact tracing)	No intervention	CEA	Cost-effectiveness model	QALYs, reduced potential costs associated with medical sequels	2007	EUR
8	Drabo et al.	2016	USA	HIV	Societal	Testing (expanded), test-and-treat (expanded HIV testing combined with immediate treatment) and PrEP	Status quo	CEA	Economic model following a compartmental HIV transmission model	QALYs, HIV incidence	2010; 2013	USD
9	Fogelberg et al.	2020	SWE	HPV	Societal	Alternative screening strategies	Alternative screening strategies	CEA	Microsimulation model	QALE, measured in terms of QALYs, incorporating disutility due to cervical cancer	2014	SEK to EUR
10	Gift et al.	2011	USA	Chlamydia, Gonorrhoea	Multiple (Societal, Payer, Healthcare)	Expedited partner treatment (EPT)	Unassisted standard partner referral (SR)	Not explicitly stated	Monte Carlo simulation model	QALYs	2008	USD
11	Ginsberg et al.	2020	ISR	HIV	Societal	PrEP use by MSM	No PrEP	CUA	Model (Excel-based)	DALYs	2018	USD
12	Kim et al.	2009	USA	HPV	Societal	Vaccination and cervical cancer screening in older women	Screening alone	CEA	Monte Carlo simulation model	QALYs, reductions in lifetime risk for cervical cancer	2006	USD
13	Kim & Goldie	2009	USA	HPV	Societal	Vaccination of girls and boys	screening alone; HPV vaccination of girls alone	CEA	Dynamic transmission model, incidence based models	QALYs, outcomes related to cervical disease and other cancers associated with HPV 16/18, HPV 6/11 associated genital warts, juvenile onset recurrent respiratory papillomatosis	2006	USD
14	Krauth et al.	2020	GER	Hepatitis C	Societal	HCV screening strategies (including MSM as a target group)	No screening	Not explicitly stated	Markov Model	QALYs	2015	EUR
15	Mahumud et al.	2019	AUS	HPV	Multiple (Societal, Health System)	HPV vaccination	Three different vaccine delivery strategies	CEA	Papillomavirus Rapid Interface for Modelling and Economics (PRIME) model	DALYs, LYs	2018	AUD
16	Nosyk et al.	2015	CAN	HIV	Multiple (Societal, Third-party payer)	Population-level HAART expansion (testing and treatment)	Constrained treatment access to HAART (75, 50%)	CEA	Dynamic compartmental transmission model	QALYs, HIV prevalence, incidence	2010	CAD
17	Ouellet et al.	2015	CAN	HIV	Societal	On-demand PrEP	Lifetime costs of HIV infection	CEA	Model based on clinical trial	QALYs, LYs	2012	CAD
18	Owusu-Edusei et al. [45]	2015	USA	Chlamydia	Societal	Vaccination	Various strategies of i.e. no screening, no vaccination, tailored screening	Not explicitly stated	Compartmental heterosexual transmission model	QALYs	2013	USD
19	Owusu-Edusei et al. [46]	2016	USA	Chlamydia	Societal	Opt-Out Chlamydia Testing	Risk-based screening (status quo)	Not explicitly stated	Compartmental heterosexual transmission model	QALYs	2014	USD
20	Regnier et al.	2014	USA	Gonorrhoea	Societal	Vaccination (meningococcal)	Standard of care (antibiotics)	Not explicitly stated	Decision-analysis model	QALYs	2012	USD
21	Rogoza et al.	2009	NL	HPV	Societal	Vaccination on top of screening	NCCSP only	Not explicitly stated	Markov model	QALYs, LYs	2009	EUR
22	Rossi et al.	2013	CAN	Hepatitis B	Societal	Universal vaccination, screening & vaccination, screening & treatment, combined screening	No targeted screening or vaccination	CEA	Markov model (decision-tree)	QALYs, HBV-associated morbidity and mortality	2011	CAD
23	Rours et al.	2016	NL	Chlamydia	Societal	Antenatal screening	No screening	CEA	Decision-analysis model	QALYs, pregnancy outcomes averted	2009	EUR
24	Van Luenen et al.	2019	NL	HIV	Societal	Guided Internet-based intervention	Attention only	CUA	Trial	QoL, QALYs	2017	EUR
25	Van Wifferen et al.	2021	NL	Chlamydia, Gonorrhoeae	Societal	Screening strategies 6 monthly	Screening strategies 3 monthly (current practice)	CEA	Dynamic infection model	QALYs, prevalence of chlamydia and gonorrhoea	2018	EUR
26	Wijnen et al.	2019	NL	HIV	Societal	Adherence Improving Self-management Strategy in HIV Care	Treatment as usual	CEA, CUA	Markov Model	QALYs	2013	EUR
27	Wolff et al.	2018	SWE	HPV	Multiple (Societal, Healthcare)	Sex-neutral vaccination	Girls-only vaccination	Not explicitly stated	Epidemiological model; Dynamic compartmental model (HPV-related cancers)	QALYs	2018	EUR
28	Zechmeister et al.	2009	AT	HPV	Multiple (Societal, Public payer)	Vaccination in addition to screening for girls only Vaccination in addition to screening for girls and boys	Screening only	CEA	Dynamic transmission model	LYG	2007	EUR
29	Zulliger et al.	2017	USA	HIV	Multiple (Societal, Payer)	HIV testing and linkage to care	5 main testing strategies	CUA	Trial	QALYs	2013	USD

*AT* Austria, *CAN* Canada, *GER* Germany, *ISR* Israel, *NL* Netherlands, *SWE* Sweden, *USA* United States of America

*AUD* Australian Dollar

*CEA* Cost-effectiveness analysis

*CUA* Cost-utility analysis

*DALY(s)* Disability-adjusted life year(s)

*EUR* Euro, *CAD* Canadian Dollar, *USD* United States Dollar

*HAART* Highly Active Antiretroviral Therapy

*HIV* Human Immunodeficiency Virus

*HPV* Human Papillomavirus

*LY(s)* Life year(s)

*LYG(s)* Life year(s) gained

*MISCAN* Microsimulation Screening Analysis

*MSM* Men Having Sex With Men

*NCCSP* National Cervical Cancer Screening Program

*NR* Not reported

*PrEP* Pre-Exposure Prophylaxis

*QALE* Quality-Adjusted Life-Expectancy

*QALY(s)* Quality-Adjusted Life Year(s)

*QoL* Quality of Life

*SEK* Swedish Krona

*SIRS* Susceptible-Infectious-Recovered-Susceptible

*This was based on the study authors’ reporting of a CEA or CUA

The types of interventions varied widely. Vaccination and screening interventions dominated, and involved stand-alone vaccination [45, 50–52], vaccination in addition to screening [40, 53–56], vaccination of girls and boys [43, 47], sex-neutral vaccination [49], screening (stand-alone) [41, 57–59], screening in addition to testing [60] and screening after vaccination [61]. Other interventions involved test and treat interventions including PrEP (Pre-Exposure Prophylaxis) [42, 62], testing and linkage to care [37] and opt-out testing strategy [46]; expedited partner treatment [46], population-level treatment expansion [44], (on-demand) PrEP [35, 63], treatment adherence interventions [38], guided internet-based behaviour intervention [36] and financial incentives [39].

### Identification, exploration and categorisation of intersectoral costs

Different intersectoral costs were identified, relating to the following sectors: patient & family, informal care, paid labour (productivity), non-paid opportunity costs (productivity), education and consumption (Table 3).

**Table 3:** Classification of intersectoral costs included in the identified studies

Sector	Cost component/resource item	N
Patient & Family	Patient time (and travel)	4
	Travel costs/expenses	9
	Out-of-pocket costs	2
	Premature burial costs^	1
	Total*	*14*
Informal Care	Informal caregiver support (non-family)	1
	Care provided by family/friends	4
	Total*	*4*
Paid Labour (productivity)	Productivity loss due to absenteeism	2
	Productivity loss due to presenteeism	2
	Lost income	2
	Lost revenue due to unemployment rate gap	1
	Fringe benefits	1
	Early retirement	1
	Avoided future production loss	1
	Intervention-related productivity gains (cost savings)	2
	Total*	*24*
Non-paid opportunity costs (productivity)	Inability to perform non-paid work/activities i.e. domestic tasks or voluntary work	4
	*Total*	*4*
Education	School absence	1
	*Total*	*1*
Consumption	Future consumption unrelated to health	1
	*Total*	*1*
Other	–	*–*

More information on the different cost components identified in each individual study can be found in supplemental file 5

N=Number of studies that captured the specific cost component(s)/resource item(s)

*Some studies captured multiple cost components/resource items in the same sector, in which case the number of studies is lower than the number would be when adding up N for each cost component/resource item in the same sector

^Premature burial costs were defined as ‘the discounted value of burial costs of the person dying from AIDS less the discounted burial costs of dying in the future from causes other than AIDS’. It was not clear where these costs incurred but in this review it was assumed that they were borne by patients/families

#### Patient & family

Patient and family costs were captured in 14 studies and included patient time, travel expenses, out-of-pocket costs and premature burial costs. Four studies estimated patient time and travel to seek care as part of healthcare costs (i.e. screening, treatment, vaccination) [43, 53, 54, 60]; although it was not clear whether this time was equated to lost productivity. Nine studies included travel costs or expenses paid for by patients/families in their analyses [35, 37, 43, 48, 50, 54, 55, 59, 61]. Most of the studies evaluated travel costs associated with the intervention being evaluated, though this was not made explicit in all studies. Studies were also not always explicit about whether this referred to travel time or financial expenses such as travel fares. Out-of-pocket costs related to costs paid for by patients/families and was accounted for by two studies [39, 56]. Premature burial costs were considered in one study [35].

#### Informal care

Caregiver support (non-family) and unpaid help by family/friends was captured in four studies [38, 42, 52, 56]. Two focused on informal care costs related to HIV/AIDS care [38, 42], one on caregiver time loss during treatment for cervical cancer patients [52] and one estimated the time taken by family members during patients’ palliative care due to hepatitis B-related cancer [56].

#### Paid labour (productivity)

Productivity costs in terms of paid labour losses were assessed in 24 studies. The majority of studies measured these in terms of absenteeism (time off work). Of those, one study estimated productivity losses in a sensitivity analysis only [49]. Two of the studies measured productivity in utilities and captured these in quality-adjusted life year (QALY) estimates [42, 56]. Here, productivity was attributable to the HIV-related morbidity and mortality and lost income due to death or disability from hepatitis B-related sequelae, both chronic conditions. Presenteeism was only accounted for by two studies [36, 58]. Few studies estimated lost income [39, 51], lost revenue due to unemployment rate gap [63], fringe benefits [39], early retirement [58], avoided future production loss (in a sensitivity analysis) [62] and intervention-related productivity gains [44, 51].

#### Non-paid opportunity costs (productivity)

Only four studies explicitly reported capturing the costs associated with non-paid work (i.e. domestic tasks, voluntary work) [36, 38, 39, 52]. It was not clear if/how many studies equated non-paid opportunity costs (i.e. lost leisure time) to lost work hours (labour).

#### Education

School absence was captured in one study only [38]. It refers to an individual missing out on potential productivity and educational attainment, but no further characteristics were stated for those who missed school. School absence was calculated by adding a unit price (based on the Dutch minimum wage) per hour missed.

#### Consumption

Future consumption costs unrelated to health were considered by one study [39]. These consumption costs referred to national average age-specific expenditures outside of healthcare and were based on the U.S. Census Consumer Expenditures Survey. The study did not further specify what this entailed.

### The impact of intersectoral costs on the study results

All studies that applied a societal perspective in addition to a healthcare and/or payer perspective presented more favourable cost-effectiveness results under the societal perspective (Table 4). Four studies reported that interventions were cost-saving from a societal perspective, whereas they were ‘only’ cost-effective under a healthcare or payer perspective [39, 40, 44, 64]. Two studies found the incremental cost-effectiveness ratios (ICERs) of their interventions decreased when applying a societal perspective in addition to a healthcare or payer perspective [47, 49]. One study found their intervention to be cost-effective from both the health system and societal perspective [52].

**Table 4:** Comparison of cost-effectiveness results from a healthcare or payer perspective and societal perspective

Authors	Intervention	Perspectives	Cost-effectiveness results from a healthcare or payer perspective	Cost-effectiveness results from a societal perspective
Adamson et al.	Financial incentives for HIV viral suppression	Societal, Healthcare	Intervention: Cost-effective US$ 49,877/QALY [US$ in 2021: 53,819]	Intervention: Cost-saving (dominant) Threshold used: range from $50,000 to $150,000 per QALY gained
			Excluding productivity and non-health care expenditures, financial incentives for viral suppression [intervention] cost US$ 3033 more per patient compared to the standard-of-care cost [comparator] (US$ 487,993 vs. US$ 484,961) [US$ in 2021: 526,562 vs. 523,290]	The total discounted lifetime societal cost was US$ 4210 lower for financial incentive patients [intervention] than for the standard-of-care patients [comparator] (US$ 268,255 vs. US$ 272,464 per patient, respectively) [US$ in 2021: 289,457 vs. 293,998]
			The greatest change among cost categories was the US$ 3685 per patient increase in lifetime ART drug costs for financial incentives compared to standard of care [US$ in 2021: 3976]	A majority of financial incentive cost savings were attributable to lifetime productivity gains of US$ 10,686 per patient. [US$ in 2021: 11,530]
			Excluding non-health care costs and productivity, financial incentives for viral suppression were cost-effective with an ICER of US$ 49,877 per QALY gained compared to the standard of care [US$ in 2021: 53,819]	Financial incentives for viral suppression gained 0.06 QALYs per patient and avoided US$ 4210 per patient compared to the standard of care (Table 2). [US$ in 2021: 4543] Financial incentives “dominated” the standard of care because patients and partners had better health outcomes for a lower cost.
			NA	Lifetime productivity gains of US$ 10,686 per patient [US$ in 2021: 11,530]
Damm et al.	HPV vaccination in addition to screening	Societal, Healthcare	Intervention (2-dose): Cost-effective 19,450€ per QALY for the bivalent [US$ in 2021: 27,305] 3645€ per QALY for the quadrivalent vaccine [US$ in 2021: 5117]	Intervention (2-dose): Cost-saving Threshold used: €50,000 Under certain scenarios: A 2-dose approach using the quadrivalent vaccine was a cost-saving strategy while using the bivalent vaccine resulted in an ICER of 13,248€ per QALY [US$ in 2021: 18,598]
			Intervention (3-dose): ICERs of a 3-dose schedule were 53,807€ per LY and 34,249€ per QALY for the bivalent vaccine [US$ in 2021: 75,539 and 48,082] and 30,910€ per LY and 14,711€ per QALY for the quadrivalent vaccine [US$ in 2021: 43,394 and 20,653]	Intervention (3-dose): Inclusion of indirect costs decreased the ICERs to 28,047€ and 8984€ per QALY for the bivalent and the quadrivalent vaccine, respectively. [US$ in 2021: 39,375 and 12,613]
				Sensitivity analysis: In scenarios with low coverage, the use of the quadrivalent vaccine led to cost savings from a societal perspective
Gift et al.	Expedited partner treatment (EPT) for Chlamydia and Gonorrhoea	Societal, Healthcare, Individual payer	Intervention (individual payer perspective): Cost-effective (under a wide range of assumptions) When EPT was not cost saving from the individual payer perspective, the incremental cost per QALY gained through EPT compared with Standard Referral (SR) was less than US$ 13,000 a cost per QALY that is typically considered to be very cost-effective [US$ in 2021: 16,124]	Intervention (societal or healthcare perspective): Cost saving Threshold used: NR It resulted in more partners treated at lower cost
Mahumud et al.	HPV vaccination	Societal, Health System	Intervention: Cost-effective From the health system and societal perspectives, the 9vHPV vaccination was very cost-effective in comparison with the status quo, with an ICER of A$47,008 and A$44,678 per DALY averted, respectively	Intervention: Cost-effective Threshold used: heuristic cost-effectiveness threshold as defined by the WHO Commission on Macroeconomics and Health (A$73,267) From the health system and societal perspectives, the 9vHPV vaccination was very cost-effective in comparison with the status quo, with an ICER of A$47,008 and A$44,678 per DALY averted, respectively
Nosyk et al.	HIV Population-level HAART expansion (testing and treatment)	Societal, Third-party payer (TPP)	Intervention: Cost-effective From a TPP perspective, ‘observed HAART access’ cost CAN$ 23,679 per QALY gained, compared to the ‘75% observed access’ scenario, and CAN$ 24,250 per QALY gained compared to the ‘50% observed access’ scenario, making observed HAART scale-up highly cost-effective [US$ in 2021: 22,625 and 23,171]	Intervention: Cost-saving Threshold used: WHO thresholds for cost-effectiveness Observed HAART access resulted in savings of CAN$ 25.1 M and CAN$ 66.5 M in present value compared to 75 and 50% HAART access scenarios, respectively [US$ in 2021: 23,955,214 and 63,467,001] Productivity gains due to HAART access more than offset the additional costs of treatment, resulting in ‘Observed HAART access’ being a dominant strategy (lower total costs, higher QALY gains)
Wolff et al.	Sex-neutral HPV vaccination	Societal, Healthcare	Intervention: Likely to be cost-effective ICER was higher from a healthcare perspective, which did not include gains from decreased production losses: 40,821€ [US$ in 2021: 50,461]	Intervention: Likely to be cost-effective Threshold used: €50,000 ICER was lower from a societal perspective, which considered cost of production loss: 38,237€ [US$ in 2021: 47,265]
Zechmeister et al.	HPV vaccination in addition to screening	Societal, Public payer	Intervention: Not cost-effective Applying a shorter time frame and a payer’s perspective or vaccinating boys may not be cost-effective without reducing the vaccine price	Intervention: Cost-effective Threshold used: NR HPV-vaccination for girls should be cost-effective when adopting a longer time-horizon and a societal perspective
			Discounted ICER for HPV-vaccination of girls only was 64,000€ per LYG [US$ in 2021: 79,111]	Discounted ICER for HPV-vaccination of girls only was 50,000€ per LYG (lower compared to a healthcare perspective) [US$ in 2021: 61,800]
			For vaccinating girls and boys compared to girls only, the corresponding ICERs were 311,000€ per LYG [US$ in 2021: 384,399]	For vaccinating girls and boys compared to girls only, the corresponding ICERs were 299,000€ per LYG (lower compared to a healthcare perspective) [US$ in 2021: 369,564]
Zulliger et al.	HIV testing and linkage to care among men having sex with men (MSM)	Societal, Payer	NA [*Results for payer and societal perspective were not reported separately*]	Intervention (venue-based testing program in all cities): Cost-saving Threshold used: $100,000 (The cost-saving threshold for HIV testing was $20,645 per new HIV diagnosis) Cost-utility analysis of the MSM Testing Initiative (MTI) programs demonstrated that all venue-based testing programs were cost-saving
				Intervention (voluntary counselling and testing strategies, social network strategies): Partially not cost-effective, depending on the city

*CAN* Canadian Dollar, *HAART* Highly Active Antiretroviral Therapy, *HIV* Human Immunodeficiency Virus, *HPV* Human Papillomavirus, *ICER* Incremental Cost-Effectiveness Ratio, *LY(G)* Life Year (Gained),* QALY* Quality-Adjusted Life Year, *US$* United States Dollar

## Discussion

This study is the first to systematically review economic evaluations of interventions relating to STIs and explore and categorise the different types of intersectoral costs captured under a societal perspective. It also presents evidence that the inclusion of intersectoral costs has an impact on the overall study results.

### Principal findings

This review found that the identified studies took a rather narrow approach to the societal perspective and only considered costs relating to a limited range of non-health sectors. For the majority of studies this meant primarily estimating paid labour losses. For others, this meant the inclusion of patient and family costs in their analyses. Very few studies considered informal care costs and other non-paid opportunity costs. Only one study included educational costs and another captured non-medical consumption costs. These findings indicate that even where a societal perspective is adopted, this may often be limited in scope, potentially omitting relevant costs from other sectors. The theoretical definition of a societal perspective, however, does not limit the potential scope to the aforementioned sectors [65].

Even though the inclusion of intersectoral costs was limited to a few cost sectors, where intersectoral costs were accounted for, this resulted in more favourable cost-effectiveness estimates.

#### Methodological challenges to the study perspective

A primary reason for studies applying a narrow societal perspective could be the methodological challenges associated with capturing these wider costs such as with data collection processes or unavailable data [13]. The identification, measurement and valuation of intersectoral costs and benefits in economic evaluations is recognised as one of four methodological challenges when assessing public health interventions [66]. This review highlights that despite methodological difficulties, it is important to be transparent and if a narrower societal perspective is applied, this needs to be explained and justified.

#### Classification of costs

This review’s cost classification scheme was established to assess whether (or not) and to what extent intersectoral costs were considered and reported explicitly and transparently. The findings suggest that there is considerable scope for exploring other wider societal costs in relation to interventions addressing STIs. This would help improve understanding of the wider societal impacts of STI-related interventions and inform the design of future, more comprehensive economic evaluations.

#### Informal care

This review shows that informal care was rarely captured in the evaluations, but where it was considered, it related to chronic conditions including HIV/AIDS, HPV-related cervical cancer and hepatitis B-related cancer. Where (long-term) care is provided informally this makes the inclusion of such costs in economic evaluations crucial. If informal care is not considered (or discussed as a study limitation) this can omit important information and underestimate the total cost burden. Future research is needed to further investigate informal care costs related to STIs, particularly those that can have chronic impacts (i.e. HIV/AIDS, hepatitis B) and those with severe long-term sequelae (i.e. pelvic inflammatory disease, chronic pelvic pain).

#### Non-paid opportunity costs

This review also found that the costs associated with unpaid work remained largely excluded from economic evaluations relating to STIs. This was difficult to judge as not all studies were explicit about which cost components/resource items they accounted for when referring to productivity costs – i.e. paid labour, volunteering or household work. A number of study authors were approached to clarify whether productivity losses accounted for paid or unpaid labour, or both. The majority responded that only paid labour losses were included due to missing data or the methodological challenges of including unpaid productivity. These findings suggest that greater transparency is needed when a societal perspective is adopted to clarify which costs and benefits are included/excluded and the justification for these decisions.

#### Education

School absence was only captured in one study. Absence from school due to an STI or seeking treatment for an STI can refer to potential productivity loss or loss of educational attainment. The study that captured absence from school valued each hour missed at school based on the national minimum wage, as informal care and domestic activities. No other costs relating to education were identified.

#### Consumption

Where future consumption costs unrelated to health were accounted for it was not clear what this involved. Examples of non-medical consumption costs can include travel expenditures or future costs for housing and food [67]. This adds to the call from this review that more transparency is needed in economic evaluations, in particular on the different cost components included (or not included), to increase consistency in terms of the costs captured and improve comparability of results across studies.

#### Distinct health impacts

Other potentially important distinct health impacts can include costs in the reproductive health and mental health sphere. However, these impacts were not captured in the selected studies. The prevention of STIs can reduce the risk of cervical cancer, pelvic inflammatory disease and infertility among women [68], which is often related to their sexual, reproductive and psychological health [69]. This implies there might be intangible costs related to STIs and their sequelae such as pain, anxiety and psychological suffering that could have an impact on people’s overall quality of life and contribute to the cost burden. Research shows that intangible costs could potentially outweigh healthcare costs and its inclusion in economic evaluations potentially result in more favourable cost-effectiveness estimates [23]. This study acknowledges there are difficulties associated with measuring and valuing intangible costs and the demonstration of attribution, and more research is needed in this area.

### Comparison to other literature

Relatively few economic evaluations related to STIs have adopted a societal perspective. This is in line with recent findings by Bloch and colleagues [70] who assessed how costs and outcomes are measured in economic evaluations relating to interventions to control STIs. Their study revealed that multiple studies did not adopt a broader perspective to account for outcomes beyond health, despite national recommendations advocating to do so [70]. The present review focused on those economic evaluations that did adopt a societal perspective and demonstrated that often this perspective is limited to certain cost sectors, predominantly the labour sector. Kim and colleagues similarly found that the CEAs they considered rarely captured impacts on sectors outside health, but if so, productivity losses were the most commonly estimated [18]. Krol and colleagues’ [71] findings also show that economic evaluations tend to predominantly assess paid labour costs [19]. Unpaid work, in comparison, has tended to receive little attention [71]. The present review confirms that non-paid work is almost entirely ignored, or not explicitly reported, in economic evaluations. As indicated above, sexual health is closely related to other sectors, including education. In 2010, Shepherd and colleagues found that school-based behavioural interventions for the prevention of STIs can improve knowledge and increase self-efficacy [72]. Research by Chong and colleagues [73] showed that online sexual-health education have an impact on an individual’s knowledge and attitudes. Overall, it is evident that public health issues and interventions can impact other sectors of society, and that the application of a societal perspective is important. This has recently been highlighted using COVID-19 as an example and demonstrating the broader societal impacts of such disease on other sectors outside health [74].

### Policy implications

A societal perspective is generally recommended to allow for all relevant costs and benefits to be considered and for an economic evaluation to be as comprehensive as possible. However, where economic studies adopt a societal perspective, but this only includes certain costs in certain sectors, relevant societal implications may be ignored. As a result, decisions based on an analysis with a limited scope might not be optimal [18]. As shown in this review, adopting a societal perspective and capturing intersectoral costs relating to STIs resulted in more favourable cost-effectiveness estimates [75]. Again, where diseases such as STIs can be prevented, treated or managed this can have an impact on an individual’s physical, mental and social health and wellbeing, their productivity as well as wider society [75]. In order to improve information communicated to policy/decision-makers all potentially relevant intersectoral costs need to be included in analyses, and if a narrow societal perspective is adopted, the exclusion of relevant costs needs to be made transparent and justified.

### Implications for research

The costs considered and included under a societal perspective differed across studies, resulting in heterogeneity of study results. This highlights that there is a need for a clear understanding of which costs were included and excluded under any perspective when reviewing and synthesising the existing literature, or when combining results from different studies undertaken in different settings. This is particularly important because the different elements of costs (i.e. care practices, wages) can differ between countries and time points. When researchers adopt data from the existing literature for use in their own work, they need to carefully assess what costs were captured before the results can be relied on and utilised.

### Strengths and limitations

The main strength of this review is that it followed a structured and rigorous process. To our knowledge, this is the first review in sexual health to apply a cost classification scheme in order to explore and categorise the intersectoral costs considered by sector. The classification scheme provides a valuable foundation for the critical appraisal of economic evaluations, in particular with regard to the consideration and identification of societal costs. The results of this study can inform the design of future, more comprehensive economic evaluations of public health interventions, building on the classification scheme presented. Another key strength of this review is the exhaustive search strategy that was developed in cooperation with an information specialist, searching nine databases and a wide range of key search terms relating to sexual health. It is however possible that some relevant search terms may have been missed. In addition, the update of the review focused on Medline only, which may have resulted in some studies being omitted, although this was mitigated by extensive hand searching. A potential weakness of the review is that because of the high volume of studies identified in the databases, an initial screening was undertaken to exclude studies where the abstract suggested that the study adopted a healthcare perspective only. This means that relevant studies could have been missed. Future research could review economic analyses that adopted a healthcare (system) perspective to assess in detail which costs these studies captured, i.e. direct medical costs, patient costs, or other costs. Another limitation of this review is that it focused on OECD member countries. Reviewing studies in non-OECD member countries could have identified other potentially relevant costs associated with interventions relating to STIs. Further, this review focused on STIs that are sexually transmitted and interventions related to infections transmitted other than sexually, could have revealed additional cost sectors.

### Future research

Further research is needed to investigate wider intersectoral costs related to STIs that were not (sufficiently) captured in this review but that could be important to inform policy/decision-making. Such research could also help explore the intersectoral costs relating to other sexual health aspects beyond disease such as sexuality, sexual behaviour, and related areas. Given the complexity of sexual health future research could explore wider intersectoral costs relating to STIs that have been considered outside of the health economics literature such as in educational journals or journals relating to social services. Furthermore, future research could explore in greater depth the distinction between intersectoral consequences associated with the disease and the intersectoral costs incurred by the intervention being evaluated, as this was not always fully clear.

## Conclusion

This systematic review suggests that economic evaluations of interventions relating to STIs that adopt a societal perspective tend to be limited in scope. This risks omitting potentially relevant intersectoral costs that could be important information for policy/decision-making. There is an urgent need for economic evaluations to be more comprehensive in order to allow policy/decision-makers to make better informed decisions.

## Supplementary Information

**Additional file 1.**

**Additional file 2.**

**Additional file 3.**

**Additional file 4.**

**Additional file 5.**

## Data Availability

All data generated or analysed during this study are included in this published article [and its supplementary information files].

## Abbreviations

AIDS: Acquired Immunodeficiency Syndrome
CBA: Cost-Benefit Analysis
CEA: Cost-Effectiveness Analysis
CHEC: Consensus on Health Economic Criteria checklist
CRD: Centre for Review and Dissemination
CUA: Cost-Utility Analysis
EUR: Euro
HIV: Human Immunodeficiency Virus
HPV: Human Papillomavirus
HSV: Herpes Simplex Virus
ICER: Incremental Cost-Effectiveness Ratio
OECD: Organisation for Economic Co-operation and Development
PrEP: Pre-Exposure Prophylaxis
PRISMA: Preferred Reporting Items for Systematic Reviews and Meta-Analyses
PROSPERO: The International Prospective Register of Systematic Reviews
QALY: Quality Adjusted Life Year
STI: Sexually Transmitted Infection
USA: United States of America
USD: United States Dollar
