# Supplementary material for: Are intersectoral costs considered in economic evaluations of interventions relating to sexually transmitted infections (STIs)? A systematic review
Source: BMC Public Health. 2022 Nov 25;22:2180. doi: 10.1186/s12889-022-14484-z (PMC9701033; doi:10.1186/s12889-022-14484-z)
Supplement: Supplementary file 2 — Additional file 2. [file 12889_2022_14484_MOESM2_ESM.docx]

Supplemental file 2: Search strategies

# PubMed (MEDLINE)

[24 May 2019]

((((((((("Sexual Behavior"[MeSH] OR sexual behavior*[tiab] OR sexual behaviour*[tiab] OR sexual activit*[tiab] OR sexual education[tiab] OR sex education[tiab] OR sexuality education[tiab] OR sexual health[tiab] OR "Sexual Health"[MeSH] OR safe sex[tiab] OR unsafe sex[tiab] OR "Unsafe Sex"[MeSH] OR "Contraception"[MeSH] OR contracepti*[tiab] OR birth control[tiab]) OR ("Sexually Transmitted Diseases"[MeSH] OR sexually transmitted disease*[tiab] OR sexually transmitted infection*[tiab] OR STD[tiab] OR STDs[tiab] OR STI[tiab] OR STIs[tiab] OR venereal disease*[tiab] OR "Acquired Immunodeficiency Syndrome"[MeSH] OR acquired immune deficiency syndrome*[tiab] OR Acquired Immunodeficiency Syndrome*[tiab] OR acquired immuno deficiency syndrome*[tiab] OR Acquired Immunologic deficiency Syndrome*[tiab] OR AIDS[tiab] OR herpes genitalis[tiab] OR genital herpes[tiab] OR "Syphilis"[MeSH] OR syphilis[tiab] OR "Chlamydia"[MeSH] OR "Chlamydia infections"[MeSH] OR chlamydia[tiab] OR "HIV"[MeSH] OR HIV[tiab] OR human immunodeficiency virus*[tiab] OR "Gonorrhea"[MeSH] OR gonorrh*[tiab] OR "Trichomonas Infections"[MeSH] OR trichomonas infection*[tiab] OR trichomonias*[tiab] OR "Pelvic Inflammatory Disease"[MeSH] OR pelvic inflammatory disease*[tiab] OR inflammatory pelvic disease*[tiab] OR HPV[tiab] OR human papillomavirus*[tiab] OR condylomata acuminata[tiab] OR genital wart*[tiab] OR venereal wart*[tiab] OR "Hepatitis B"[MeSH] OR "Hepatitis B"[tiab])) AND ((cost[tiab] OR costs[tiab] OR "costs and cost analysis"[MeSH:noexp] OR (cost benefit analyses[Tiab] OR cost benefit analys*[Tiab]) OR "cost-benefit analysis"[MeSH] OR "health care costs"[MeSH:noexp]) OR (("Cost of Illness"[MeSH] OR (health expenditure[tiab] OR health expenditure*[tiab]))))))))

NOT (developing countr*[Title] OR third world[Title] OR underdeveloped countr*[Title] OR under developed countr*[Title] OR "Latin America"[Mesh] OR "Africa"[MeSH] OR "Asia, Northern"[ MeSH] OR "Asia, Central"[MeSH] OR "Asia, Southeastern"[MeSH] OR "Asia, Western"[MeSH] OR "Central America"[MeSH] OR "Caribbean Region"[MeSH] OR Latin America[Title] OR Africa[Title] OR Central America[Title] OR Caribbean Region[Title] OR "Sensory Aids"[Mesh] OR hearing aids[Title] OR sensory aids[Title]))))

NOT (Afghanistan[Title] OR Africa[Title] OR Albania[Title] OR Algeria[Title] OR Angola[Title] OR Antigua[Title] OR Argentina[Title] OR Armenia[Title] OR Azerbaijan[Title] OR Bangladesh[Title] OR Barbados[Title] OR Barbuda[Title] OR Belarus[Title] OR Belize[Title] OR Brazil[Title] OR Bhutan[Title] OR Bolivia[Title] OR Bosnia[Title] OR Botswana[Title] OR Bulgaria[Title] OR Burkina Faso[Title] OR Burundi[Title] OR Cambodia[Title] OR Cameroon[Title] OR Central African Republic[Title] OR Chad[Title] OR Colombia[Title] OR Comoros[Title] OR Congo[Title] OR Costa Rica[Title] OR Croatia[Title] OR Cuba[Title] OR Czech*[Title] OR Congo[Title] OR Djibouti[Title] OR Dominica[Title] OR Dominican[Title] OR East Timor[Title] OR Ecuador[Title] OR Egypt[Title] OR El Salvador[Title] OR Equatorial Guinea[Title] OR Eritrea[Title] OR Estonia[Title] OR Ethiopia[Title] OR Fiji[Title] OR Gabon[Title] OR Gambia[Title] OR Ghana[Title] OR Grenada[Title] OR Guatemala[Title] OR Guinea-Bissau[Title] OR Guyana[Title] OR Haiti[Title] OR Honduras[Title] OR Hungary[Title] OR India[Title] OR Indonesia[Title] OR Iran[Title] OR Iraq[Title] OR Ivory Coast[Title] OR Jamaica[Title] OR Jordan[Title] OR Kazakhstan[Title] OR Kenya[Title] OR Kiribati[Title] OR Kyrgyzstan[Title] OR Laos[Title] OR Latvia[Title] OR Lebanon[Title] OR Lesotho[Title] OR Liberia[Title] OR Libya[Title] OR Lithuania[Title] OR Madagascar[Title] OR Malawi[Title] OR Malaysia[Title] OR Maldives[Title] OR Mali[Title] OR Marshall Islands[Title] OR Mauritania[Title] OR Mauritius[Title] OR Micronesia[Title] OR Moldova[Title] OR Mongolia[Title] OR Montenegro[Title] OR Morocco[Title] OR Mozambique[Title] OR Myanmar[Title] OR Namibia[Title] OR Nepal[Title] OR New Guinea[Title] OR Nicaragua[Title] OR Niger[Title] OR Nigeria[Title] OR Oman[Title] OR Pakistan[Title] OR Palau[Title] OR Panama[Title] OR Papua New Guinea[Title] OR Paraguay[Title] OR Benin[Title] OR China[Title] OR Peru[Title] OR Philippines[Title] OR Poland[Title] OR Cape Verde[Title] OR Georgia[Title] OR Kosovo[Title] OR Macedonia[Title] OR Yemen[Title] OR Romania[Title] OR Russia[Title] OR Rwanda[Title] OR Saint Kitts[Title] OR Saint Vincent[Title] OR Saint Lucia[Title] OR Sao Tome Principe[Title] OR Saudi Arabia[Title] OR Senegal[Title] OR Serbia[Title] OR Seychelles[Title] OR Sierra Leone[Title] OR Slovak*[Title] OR South Africa[Title] OR Solomon Islands[Title] OR Somalia[Title] OR Sri Lanka[Title] OR Sri-Lanka[Title] OR Sudan[Title] OR Suriname[Title] OR Swaziland[Title] OR Syria[Title] OR Tajikistan[Title] OR Tanzania[Title] OR Thailand[Title] OR Togo[Title] OR Tonga[Title] OR Trinidad[Title] OR Tobago[Title] OR Tunisia[Title] OR Turkey[Title] OR Turkmenistan[Title] OR Uganda[Title] OR Ukraine[Title] OR Uruguay[Title] OR Uzbekistan[Title] OR Vanuatu[Title] OR Venezuela[Title] OR Vietnam[Title] OR Samoa[Title] OR Zambia[Title] OR Zimbabwe[Title]))

Note: The term "cost-benefit analysis"[MeSH], which (among other) searches for the following terms: Cost Effectiveness; Effectiveness, Cost; Cost-Effectiveness Analysis; Analysis, Cost-Effectiveness; Cost Effectiveness Analysis.

# EMBASE (via Ovid)

[24 May 2019]

1. sexual health/

2. sexual education/

3. (sexual behaviour or sexual behaviour).kw.

4. safe sex.kw.

5. unsafe sex.kw.

6. (contraception or contraceptions or contraceptive or contraceptives or birth control).kw.

7. exp Sexually Transmitted Diseases/

8. (STIs or STI or STDs or STD or venereal disease or venereal diseases).kw.

9. (AIDS or acquired immun* deficiency syndrome or human immunodeficiency virus).ti,ab,kw.

10. (HIV or human immun* deficiency virus or human immunodeficiency virus).ti,ab,kw.

11. (Herpes Genitalis or genital herpes or genital infection or genital infections).ti,ab,kw.

12. Syphilis.ti,ab,kw.

13. Chlamydia.ti,ab,kw.

14. Gonorrh*.ti,ab,kw.

15. (trichomonas or trichomoniasis).ti,ab,kw.

16. Pelvic Inflammatory Disease*.ti,ab,kw.

17. (HPV infection* or Papillomavirus Infection or Papillomavirus Infections).ti,ab,kw.

18. (Genital wart or genital warts or venereal wart or venereal warts or condylomata acuminata).ti,ab,kw.

19. Hepatitis B.ti,ab,kw.

20. cost of illness/

21. (healthcare cost* or health care cost*).kw.

22. (cost or costs).tw.

23. 20 or 21 or 22

24. exp sensory aid/ or exp hearing aids/

25. ("sensory aid*" or "hearing aid*").ti,ab,kw.

26. 24 or 25

27. exp developing country/ or exp Africa/ or exp Caribbean/ or exp Central America/ or exp Southeast Asia/ or exp USSR/ or exp Melanesia/ or exp "Federated States of Micronesia"/ or exp Polynesia/ or exp French Polynesia/ or exp Atlantic islands/ or exp Indian Ocean/ or exp Central Africa/

28. "Macedonia (republic)"/ or Russian Federation/ or China/ or Afghanistan/ or Albania/ or Bahrain/ or Belarus/ or Brazil/ or Bhutan/ or Bolivia/ or "Federation of Bosnia and Herzegovina"/ or "Bosnia and Herzegovina"/ or Bulgaria/ or Congo/ or Ecuador/ or Guyana/ or "Punjab (India)"/ or India/ or Iran/ or Iraq/ or Jordan/ or Kuwait/ or Lebanon/ or Moldova/ or Mongolia/ or Montenegro/ or Nepal/ or Oman/ or Pakistan/ or Paraguay/ or Puerto Rico/ or Peru/ or Philippines/ or Kosovo/ or Yemen/ or Romania/ or Saudi Arabia/ or Serbia/ or Sri Lanka/ or Suriname/ or Syria/ or Uruguay/ or Venezuela/ or Qatar/ or United Arab Emirates/ or Colombia/

29. (Afghanistan or Africa or Albania or Algeria or Angola or Antigua or Argentina or Armenia or Azerbaijan or Bangladesh or Barbados or Barbuda or Bahrain or Belarus or Belize or Brazil or Bhutan or Bolivia or Bosnia or Botswana or Bulgaria or Burkina Faso or Burundi or Cambodia or Cameroon or Chad or Comoros or Congo or Costa Rica or Croatia or Cuba or Congo or Djibouti or Dominica or Dominican or East Timor or Ecuador or Egypt or El Salvador or Equatorial Guinea or Eritrea or Ethiopia or Fiji or Gabon or Gambia or Ghana or Grenada or Guatemala or Guinea or Guyana or Haiti or Honduras or India or Indonesia or Iran or Iraq or Jamaica or Jordan or Kazakhstan or Kenya or Kiribati or Kyrgyzstan or Kuwait or Laos or Lebanon or Lesotho or Liberia or Libya or Madagascar or Malawi or Malaysia or Maldives or Mali or Mauritania or Mauritius or Micronesia or Moldova or Mongolia or Montenegro or Morocco or Mozambique or Myanmar or Namibia or Nepal or Nicaragua or Niger or Nigeria or Oman or Pakistan or Palau or Panama or Paraguay or Benin or China or Peru or Philippines or Georgia or Kosovo or Macedonia or Yemen or Romania or Russia or Rwanda or Saint Kitts or Saint Vincent or Saint Lucia or Sao Tome Principe or Saudi Arabia or Senegal or Serbia or Seychelles or Sierra Leone or South Africa or Solomon Islands or Somalia or Sri Lanka or Sri-Lanka or Sudan or Suriname or Swaziland or Syria or Tajikistan or Tanzania or Thailand or Togo or Tonga or Trinidad or Tobago or Tunisia or Turkmenistan or Uganda or Ukraine or Uruguay or Uzbekistan or Vanuatu or Venezuela or Vietnam or Samoa or Zambia or Zimbabwe or Qatar or United Arab Emirates or Colombia).ti.

30. 27 or 28 or 29

31. 1 or 2 or 3 or 4 or 5 or 6 or 7 or 8 or 9 or 10 or 11 or 12 or 13 or 14 or 15 or 16 or 17 or 18 or 19

32. 23 and 31

33. 32 not 26

34. 33 not 30

35. 34 not ((exp animal/ or nonhuman/) not exp human/)

36. limit 35 to ((dutch or english or german) and last 20 years)

# Web of Science Core Collection

[24 May 2019]

# 31 (#29 not #30)

# 30 (ti=("sensory aids" or "hearing aids"))

# 29 (#27 not #28)

# 28 (ti=(Afghanistan or Africa or Albania or Algeria or Angola or Antigua or Argentina or Armenia or Azerbaijan or Bangladesh or Barbados or Barbuda or Belarus or Belize or Brazil or Bhutan or Bolivia or Bosnia or Botswana or Bulgaria or Burkina Faso or Burundi or Cambodia or Cameroon or Central African Republic or Chad or Chile or Colombia or Comoros or Congo or Costa Rica or Croatia or Cuba or Czech* or Congo or Djibouti or Dominica or Dominican or East Timor or Ecuador or Egypt or El Salvador or Equatorial Guinea or Eritrea or Estonia or Ethiopia or Fiji or Gabon or Gambia or Ghana or Grenada or Guatemala or Guinea-Bissau or Guyana or Haiti or Honduras or Hungary or India or Indonesia or Iran or Iraq or Ivory Coast or Jamaica or Jordan or Kazakhstan or Kenya or Kiribati or Kyrgyzstan or Laos or Latvia or Lebanon or Lesotho or Liberia or Libya or Lithuania or Madagascar or Malawi or Malaysia or Maldives or Mali or Marshall Islands or Mauritania or Mauritius or Mexico or Micronesia or Moldova or Mongolia or Montenegro or Morocco or Mozambique or Myanmar or Namibia or Nepal or New Guinea or Nicaragua or Niger or Nigeria or Oman or Pakistan or Palau or Panama or Papua New Guinea or Paraguay or Benin or China or Peru or Philippines or Poland or Cape Verde or Georgia or Kosovo or Macedonia or Yemen or Romania or Russia or Rwanda or Saint Kitts or Saint Vincent or Saint Lucia or Sao Tome Principe or Saudi Arabia or Senegal or Serbia or Seychelles or Sierra Leone or Slovak* or South Africa or Solomon Islands or Somalia or Sri Lanka or Sri-Lanka or Sudan or Suriname or Swaziland or Syria or Tajikistan or Tanzania or Thailand or Togo or Tonga or Trinidad or Tobago or Tunisia or Turkey or Turkmenistan or Uganda or Ukraine or Uruguay or Uzbekistan or Vanuatu or Venezuela or Vietnam or Samoa or Zambia or Zimbabwe))

# 27 #26 AND #21

# 26 #25 OR #24 OR #23 OR #22

# 25 (ti=(cost* or "cost benefit analys*" or "health care cost*" or "economic evaluation"))

# 24 (ts=("cost benefit analys*" or "health care cost*" or "economic evaluation"))

# 23 (ts=("cost of illness*" or "burden of illness*" or "burden of disease*" or "economic burden"))

# 22 (ti=("cost of illness*" or "health expenditure*" or "burden of illness*" or "burden of disease*" "economic burden"))

# 21 #20 OR #19 OR #18 OR #17 OR #16 OR #15 OR #14 OR #13 OR #12 OR #11 OR #10 OR #9 OR #8 OR #7 OR #6 OR #5 OR #4 OR #3 OR #2 OR #1

# 20 (ti=(Hepatitis B))

# 19 (ti=(Genital wart* or venereal wart*))

# 18 (ti=(HPV infection* or Human Papillomavirus Infection* or Papillomavirus Infection*))

# 17 (ti=(Pelvic Inflammatory Disease*))

# 16 (ti=(trichomonas infection or trichomoniasis))

# 15 (ti=(Gonorrh*))

# 14 (ti=(Chlamydia))

# 13 (ti=(Syphilis))

# 12 (ti=(Herpes Genitalis or genital herpes))

# 11 (ti=(HIV or human immuno deficiency virus or human immunodeficiency virus or human immune deficiency virus))

# 10 (ti=(AIDS or acquired immuno deficiency syndrome or acquired immune deficiency syndrome))

# 9 (ti=(sexually transmitted infection* or sexually transmitted disease* or STI or STIs or STD or STDs or venereal disease*))

# 8 (ts=(sexually transmitted disease*))

# 7 (ts=(sexually transmitted infection*))

# 6 (ti=(contracept* or birth control))

# 5 (ti=(sexual health or sexual behavi* or sexual activit* or sex* education))

# 4 (ts=(contracept*))

# 3 (ts=(sexual behavi*))

# 2 (ts=(sexual health))

# 1 (ts=(sex* education))

For Web of Science the following indexes were searched: SCI-EXPANDED (Science Citation Index Expanded), SSCI (Social Sciences Citation Index), A&HCI (Arts & Humanities Citation Index), ESCI (Emerging Sources Citation Index). Timespan=1999-2019.

# CINAHL (EBSCO)

[26 May 2019]

Limitation Date: 19990101-20191231

Interface: EBSCOhost Research Databases

S29 S26 NOT ( MH "Animals" NOT MH "Human )

S28 S26 NOT ( MH "Animals" NOT MH "Human )

S27 S26 NOT ( MH "Animals" NOT MH "Human )

S26 S24 NOT S25 NOT S19

S25 S20 OR S21 OR S22

S24 S18 AND S23

S23 S1 OR S2 OR S3 OR S4 OR S5 OR S6 OR S7 OR S8 OR S9 OR S10 OR S11 OR S12 OR S13 OR S14 OR S15 OR S16 OR S17

S22 (MH "Developing Countries") OR ( MH "Low and Middle Income Countries" )

S21 "developing countr*" or "underdeveloped countr*" or "under developed countr*" or "third world" or "least developed countr*" or "less developed countr*" or "under developed nation*" or "developing nation*"

S20 TI (“Russia” or “China” or “Brazil” or “Brasil” or “Bosnia” or “Herzegovina” or “Kuwait” or “Moldova” or “Mongolia” or “Montenegro” or “Nepal” or “Oman” or “Pakistan” or “Paraguay” or “Puerto Rico” or “Kosovo” or “Yemen” or “Romania” or “Saudi Arabia” or “Sri Lanka” or “Suriname” or “Syria” or “Uruguay” or “Venezuela” or “Qatar” or “United Arab“ or “United Arab Emirates” or “Colombia” or “Afghanistan” OR “Africa” OR “Albania” OR “Algeria” OR “Angola” OR “Antigua” OR “Argentina” OR “Armenia” OR “Azerbaijan” OR “Bangladesh” OR “Barbados” OR “Barbuda” OR “Bahrain” OR “Belarus” OR “Belize” OR “Bhutan” OR “Bolivia” OR “Botswana” OR “Bulgaria” OR “Burkina Faso” OR “Burundi” OR “Cambodia” OR “Cameroon” OR “Chad” OR “Comoros” OR “Congo” OR “Costa Rica” OR “Croatia” OR “Cuba” OR “Congo” OR “Djibouti” OR “Dominica” OR “Dominican” OR “East Timor” OR “Ecuador” OR “Egypt” OR “El Salvador” OR “Guinea” OR “Eritrea” OR “Ethiopia” OR “Fiji” OR “Gabon” OR “Gambia” OR “Ghana” OR “Grenada” OR “Guatemala” OR “Guinea” OR “Guyana” OR “Haiti” OR “Honduras” OR “India” OR “Indonesia” OR “Iran” OR “Iraq” OR “Jamaica” OR “Jordan” OR “Kazakhstan” OR “Kenya” OR “Kiribati” OR “Kyrgyzstan” OR “Laos” OR “Lebanon” OR “Lesotho” OR “Liberia” OR “Libya” OR “Madagascar” OR “Malawi” OR “Malaysia” OR “Maldives” OR “Mali” OR “Mauritania” OR “Mauritius” OR “Morocco” OR “Mozambique” OR “Myanmar” OR “Namibia” OR “Nicaragua” OR “Niger” OR “Nigeria” OR “Palau” OR “Panama” OR “Benin” OR “Peru” OR “Philippines” OR “Georgia” OR “Macedonia” OR “Rwanda” OR “Saint Kitts” OR “Saint Vincent” OR “Saint Lucia” OR “Sao Tome Principe” OR “Senegal” OR “Serbia” OR “Seychelles” OR “Sierra Leone” OR “South Africa” OR “Solomon Islands” OR “Somalia” OR “Sudan” OR “Swaziland” OR “Tajikistan” OR “Tanzania” OR “Thailand” OR “Togo” OR “Tonga” OR “Trinidad” OR “Tobago” OR “Tunisia” OR “Turkmenistan” OR “Uganda” OR “Ukraine” OR “Uzbekistan” OR “Vanuatu” OR “Vietnam” OR “Samoa” OR “Zambia” OR “Zimbabwe”) or AB (“Russia” or “China” or “Brazil” or “Brasil” or “Bosnia” or “Herzegovina” or “Kuwait” or “Moldova” or “Mongolia” or “Montenegro” or “Nepal” or “Oman” or “Pakistan” or “Paraguay” or “Puerto Rico” or “Kosovo” or “Yemen” or “Romania” or “Saudi Arabia” or “Sri Lanka” or “Suriname” or “Syria” or “Uruguay” or “Venezuela” or “Qatar” or “United Arab“ or “United Arab Emirates” or “Colombia” or “Afghanistan” OR “Africa” OR “Albania” OR “Algeria” OR “Angola” OR “Antigua” OR “Argentina” OR “Armenia” OR “Azerbaijan” OR “Bangladesh” OR “Barbados” OR “Barbuda” OR “Bahrain” OR “Belarus” OR “Belize” OR “Bhutan” OR “Bolivia” OR “Botswana” OR “Bulgaria” OR “Burkina Faso” OR “Burundi” OR “Cambodia” OR “Cameroon” OR “Chad” OR “Comoros” OR “Congo” OR “Costa Rica” OR “Croatia” OR “Cuba” OR “Congo” OR “Djibouti” OR “Dominica” OR “Dominican” OR “East Timor” OR “Ecuador” OR “Egypt” OR “El Salvador” OR “Guinea” OR “Eritrea” OR “Ethiopia” OR “Fiji” OR “Gabon” OR “Gambia” OR “Ghana” OR “Grenada” OR “Guatemala” OR “Guinea” OR “Guyana” OR “Haiti” OR “Honduras” OR “India” OR “Indonesia” OR “Iran” OR “Iraq” OR “Jamaica” OR “Jordan” OR “Kazakhstan” OR “Kenya” OR “Kiribati” OR “Kyrgyzstan” OR “Laos” OR “Lebanon” OR “Lesotho” OR “Liberia” OR “Libya” OR “Madagascar” OR “Malawi” OR “Malaysia” OR “Maldives” OR “Mali” OR “Mauritania” OR “Mauritius” OR “Morocco” OR “Mozambique” OR “Myanmar” OR “Namibia” OR “Nicaragua” OR “Niger” OR “Nigeria” OR “Palau” OR “Panama” OR “Benin” OR “Peru” OR “Philippines” OR “Georgia” OR “Macedonia” OR “Rwanda” OR “Saint Kitts” OR “Saint Vincent” OR “Saint Lucia” OR “Sao Tome Principe” OR “Senegal” OR “Serbia” OR “Seychelles” OR “Sierra Leone” OR “South Africa” OR “Solomon Islands” OR “Somalia” OR “Sudan” OR “Swaziland” OR “Tajikistan” OR “Tanzania” OR “Thailand” OR “Togo” OR “Tonga” OR “Trinidad” OR “Tobago” OR “Tunisia” OR “Turkmenistan” OR “Uganda” OR “Ukraine” OR “Uzbekistan” OR “Vanuatu” OR “Vietnam” OR “Samoa” OR “Zambia” OR “Zimbabwe”)

S19 "sensory aid*" or "hearing aid*" or "decision aid*" or "smoking cessation aid*"

S18 "cost analys*" or "cost benefit" or "cost effectiv*" or "cost minimi*" or "cost utility" or "health cost*" or "healthcare cost*" or "direct cost*" or "indirect cost*" or "health care cost*" or "health expenditure*" or "burden of illness*" or "burden of disease*" or "economic burden" or "economic evaluation*" or "health* economic*" or "cost* of illness*" or "illness* cost*" or "cost* of disease*" or "disease* cost*" or "sickness cost*" or "productivity cost*" or "cost* and benefit*"

S17 TI ("Hepatitis B") or AB ("Hepatitis B")

S16 TI ("Genital wart*" or "venereal wart*" or "condylomata acuminata") or AB ("Genital wart*" or "venereal wart*" or "condylomata acuminata")

S15 TI ("HPV infection*" or "Human Papillomavirus Infection*") or AB ("HPV infection*" or "Human Papillomavirus Infection*")

S14 TI ("Pelvic Inflammatory Disease*") or AB ("Pelvic Inflammatory Disease*")

S13 TI ("trichomonas vagin*") or AB ("trichomonas vagin*")

S12 TI ("Gonorrh*") or AB ("Gonorrh*")

S11 TI ("Chlamydia trachomatis") or AB ("Chlamydia trachomatis")

S10 TI ("Syphilis") or AB ("Syphilis")

S9 TI ("genital herpes" or "Herpes Genitalis" or "genital infection") or AB ("genital herpes" or "Herpes Genitalis" or "genital infection")

S8 TI ("HIV" or "human immun* deficiency virus" or "human immunodeficiency virus") or AB ("HIV" or "human immun* deficiency virus" or "human immunodeficiency virus")

S7 TI ("AIDS" or "acquired immun* deficiency syndrome" or "acquired immunodeficiency syndrome") or AB ("AIDS" or "acquired immun* deficiency syndrome" or "acquired immunodeficiency syndrome")

S6 TI ( "STI" or "STIs" or "STD" or "STDs" OR “venereal disease” OR “venereal diseases” ) OR AB ( "STI" or "STIs" or "STD" or "STDs" OR “venereal disease” OR “venereal diseases” )

S5 (MH "Sexually Transmitted Diseases")

S4 (MH "Contraception")

S3 "sexual behavior" or "sexual behaviour"

S2 (MH "Sex Education")

S1 (MH "Sexual Health")

# PsycINFO (EBSCO)

[26 May 2019]

Limiters - Publication Year: 1999-2019

S26 S25 NOT (PO Animal NOT PO Human)

S25 S24 NOT S21 NOT S22

S24 S18 AND S23

S23 S1 OR S2 OR S3 OR S4 OR S5 OR S6 OR S7 OR S8 OR S9 OR S10 OR S11 OR S12 OR S13 OR S14 OR S15

S22 S19 OR S20

S21 "sensory aid*" or "hearing aid*" or "decision aid*" or “smoking cessation aid*”

S20 TI (“Russia” or “China” or “Brazil” or “Brasil” or “Bosnia” or “Herzegovina” or “Kuwait” or “Moldova” or “Mongolia” or “Montenegro” or “Nepal” or “Oman” or “Pakistan” or “Paraguay” or “Puerto Rico” or “Kosovo” or “Yemen” or “Romania” or “Saudi Arabia” or “Sri Lanka” or “Suriname” or “Syria” or “Uruguay” or “Venezuela” or “Qatar” or “United Arab“ or “United Arab Emirates” or “Colombia” or “Afghanistan” OR “Africa” OR “Albania” OR “Algeria” OR “Angola” OR “Antigua” OR “Argentina” OR “Armenia” OR “Azerbaijan” OR “Bangladesh” OR “Barbados” OR “Barbuda” OR “Bahrain” OR “Belarus” OR “Belize” OR “Bhutan” OR “Bolivia” OR “Botswana” OR “Bulgaria” OR “Burkina Faso” OR “Burundi” OR “Cambodia” OR “Cameroon” OR “Chad” OR “Comoros” OR “Congo” OR “Costa Rica” OR “Croatia” OR “Cuba” OR “Congo” OR “Djibouti” OR “Dominica” OR “Dominican” OR “East Timor” OR “Ecuador” OR “Egypt” OR “El Salvador” OR “Guinea” OR “Eritrea” OR “Ethiopia” OR “Fiji” OR “Gabon” OR “Gambia” OR “Ghana” OR “Grenada” OR “Guatemala” OR “Guinea” OR “Guyana” OR “Haiti” OR “Honduras” OR “India” OR “Indonesia” OR “Iran” OR “Iraq” OR “Jamaica” OR “Jordan” OR “Kazakhstan” OR “Kenya” OR “Kiribati” OR “Kyrgyzstan” OR “Laos” OR “Lebanon” OR “Lesotho” OR “Liberia” OR “Libya” OR “Madagascar” OR “Malawi” OR “Malaysia” OR “Maldives” OR “Mali” OR “Mauritania” OR “Mauritius” OR “Morocco” OR “Mozambique” OR “Myanmar” OR “Namibia” OR “Nicaragua” OR “Niger” OR “Nigeria” OR “Palau” OR “Panama” OR “Benin” OR “Peru” OR “Philippines” OR “Georgia” OR “Macedonia” OR “Rwanda” OR “Saint Kitts” OR “Saint Vincent” OR “Saint Lucia” OR “Sao Tome Principe” OR “Senegal” OR “Serbia” OR “Seychelles” OR “Sierra Leone” OR “South Africa” OR “Solomon Islands” OR “Somalia” OR “Sudan” OR “Swaziland” OR “Tajikistan” OR “Tanzania” OR “Thailand” OR “Togo” OR “Tonga” OR “Trinidad” OR “Tobago” OR “Tunisia” OR “Turkmenistan” OR “Uganda” OR “Ukraine” OR “Uzbekistan” OR “Vanuatu” OR “Vietnam” OR “Samoa” OR “Zambia” OR “Zimbabwe”) or AB (“Russia” or “China” or “Brazil” or “Brasil” or “Bosnia” or “Herzegovina” or “Kuwait” or “Moldova” or “Mongolia” or “Montenegro” or “Nepal” or “Oman” or “Pakistan” or “Paraguay” or “Puerto Rico” or “Kosovo” or “Yemen” or “Romania” or “Saudi Arabia” or “Sri Lanka” or “Suriname” or “Syria” or “Uruguay” or “Venezuela” or “Qatar” or “United Arab“ or “United Arab Emirates” or “Colombia” or “Afghanistan” OR “Africa” OR “Albania” OR “Algeria” OR “Angola” OR “Antigua” OR “Argentina” OR “Armenia” OR “Azerbaijan” OR “Bangladesh” OR “Barbados” OR “Barbuda” OR “Bahrain” OR “Belarus” OR “Belize” OR “Bhutan” OR “Bolivia” OR “Botswana” OR “Bulgaria” OR “Burkina Faso” OR “Burundi” OR “Cambodia” OR “Cameroon” OR “Chad” OR “Comoros” OR “Congo” OR “Costa Rica” OR “Croatia” OR “Cuba” OR “Congo” OR “Djibouti” OR “Dominica” OR “Dominican” OR “East Timor” OR “Ecuador” OR “Egypt” OR “El Salvador” OR “Guinea” OR “Eritrea” OR “Ethiopia” OR “Fiji” OR “Gabon” OR “Gambia” OR “Ghana” OR “Grenada” OR “Guatemala” OR “Guinea” OR “Guyana” OR “Haiti” OR “Honduras” OR “India” OR “Indonesia” OR “Iran” OR “Iraq” OR “Jamaica” OR “Jordan” OR “Kazakhstan” OR “Kenya” OR “Kiribati” OR “Kyrgyzstan” OR “Laos” OR “Lebanon” OR “Lesotho” OR “Liberia” OR “Libya” OR “Madagascar” OR “Malawi” OR “Malaysia” OR “Maldives” OR “Mali” OR “Mauritania” OR “Mauritius” OR “Morocco” OR “Mozambique” OR “Myanmar” OR “Namibia” OR “Nicaragua” OR “Niger” OR “Nigeria” OR “Palau” OR “Panama” OR “Benin” OR “Peru” OR “Philippines” OR “Georgia” OR “Macedonia” OR “Rwanda” OR “Saint Kitts” OR “Saint Vincent” OR “Saint Lucia” OR “Sao Tome Principe” OR “Senegal” OR “Serbia” OR “Seychelles” OR “Sierra Leone” OR “South Africa” OR “Solomon Islands” OR “Somalia” OR “Sudan” OR “Swaziland” OR “Tajikistan” OR “Tanzania” OR “Thailand” OR “Togo” OR “Tonga” OR “Trinidad” OR “Tobago” OR “Tunisia” OR “Turkmenistan” OR “Uganda” OR “Ukraine” OR “Uzbekistan” OR “Vanuatu” OR “Vietnam” OR “Samoa” OR “Zambia” OR “Zimbabwe”)

S19 "developing countr*" or "underdeveloped countr*" or "under developed countr*" or "third world" or "least developed countr*" or "less developed countr*" or "under developed nation*" or "developing nation*"

S18 S16 OR S17

S17 "cost analys*" or "cost benefit" or "cost effectiv*" or "cost minimi*" or "cost utility" or "health cost*" or "healthcare cost*" or "direct cost*" or "indirect cost*" or "health care cost*" or "health expenditure*" or "burden of illness*" or "burden of disease*" or "economic burden" or "economic evaluation*" or "health* economic*" or "cost* of illness*" or "illness* cost*" or "cost* of disease*" or "disease* cost*" or "sickness cost*" or "productivity cost*" or "cost* and benefit*"

S16 DE "Costs and Cost Analysis" OR DE "Health Care Costs" OR DE "Health Care Economics"

S15 TI ("Hepatitis B") or AB ("Hepatitis B")

S14 TI ("Genital wart" or "Genital warts" or "venereal wart" or "venereal warts" or "condylomata acuminata") or AB ("Genital wart" or "Genital warts" or "venereal wart" or "venereal warts" or "condylomata acuminata")

S13 TI ("HPV infection*" or "Human Papillomavirus Infection*") or AB ("HPV infection*" or "Human Papillomavirus Infection*")

S12 TI ("Pelvic Inflammatory Disease*") or AB ("Pelvic Inflammatory Disease*")

S11 TI ("trichomonas vagin*") or AB ("trichomonas vagin*")

S10 TI ("Gonorrh*") or AB ("Gonorrh*")

S9 TI ("Chlamydia trachomatis") or AB ("Chlamydia trachomatis")

S8 TI ("Syphilis") or AB ("Syphilis")

S7 TI ("genital herpes" or "Herpes Genitalis" or "genital infection") or AB ("genital herpes" or "Herpes Genitalis" or "genital infection")

S6 TI ("HIV" or "human immun* deficiency virus" or "human immunodeficiency virus") or AB ("HIV" or "human immun* deficiency virus" or "human immunodeficiency virus")

S5 TI ("AIDS" or "acquired immun* deficiency syndrome" or "acquired immunodeficiency syndrome") or AB ("AIDS" or "acquired immun* deficiency syndrome" or "acquired immunodeficiency syndrome")

S4 TI ("STI" or "STIs" or "STD" or "STDs" OR “venereal disease” OR “venereal diseases”) OR AB ("STI" or "STIs" or "STD" or "STDs" OR “venereal disease” OR “venereal diseases”)

S3 DE "Birth Control"

S2 DE "Sexual Health" OR DE "Sexually Transmitted Diseases"

S1 DE "Sex Education"

# EconLit (EBSCO)

[26 May 2019]

Limiters - Published Date: 19990101-20181231

S30 S26 NOT S22

S29 S26 NOT S22

S28 S26 NOT S22

S27 S26 NOT S22

S26 S24 NOT S19 NOT S25

S25 S20 OR S21

S24 S18 AND S23

S23 S1 OR S2 OR S3 OR S4 OR S5 OR S6 OR S7 OR S8 OR S9 OR S10 OR S11 OR S12 OR S13 OR S14 OR S15 OR S16 OR S17

S22 "animal" or "animals" or "nonhuman"

S21 "developing countr*" or "underdeveloped countr*" or "under developed countr*" or "third world" or "least developed countr*" or "less developed countr*" or "under developed nation*" or "developing nation*"

S20 TI (“Russia” or “China” or “Brazil” or “Brasil” or “Bosnia” or “Herzegovina” or “Kuwait” or “Moldova” or “Mongolia” or “Montenegro” or “Nepal” or “Oman” or “Pakistan” or “Paraguay” or “Puerto Rico” or “Kosovo” or “Yemen” or “Romania” or “Saudi Arabia” or “Sri Lanka” or “Suriname” or “Syria” or “Uruguay” or “Venezuela” or “Qatar” or “United Arab“ or “United Arab Emirates” or “Colombia” or “Afghanistan” OR “Africa” OR “Albania” OR “Algeria” OR “Angola” OR “Antigua” OR “Argentina” OR “Armenia” OR “Azerbaijan” OR “Bangladesh” OR “Barbados” OR “Barbuda” OR “Bahrain” OR “Belarus” OR “Belize” OR “Bhutan” OR “Bolivia” OR “Botswana” OR “Bulgaria” OR “Burkina Faso” OR “Burundi” OR “Cambodia” OR “Cameroon” OR “Chad” OR “Comoros” OR “Congo” OR “Costa Rica” OR “Croatia” OR “Cuba” OR “Congo” OR “Djibouti” OR “Dominica” OR “Dominican” OR “East Timor” OR “Ecuador” OR “Egypt” OR “El Salvador” OR “Guinea” OR “Eritrea” OR “Ethiopia” OR “Fiji” OR “Gabon” OR “Gambia” OR “Ghana” OR “Grenada” OR “Guatemala” OR “Guinea” OR “Guyana” OR “Haiti” OR “Honduras” OR “India” OR “Indonesia” OR “Iran” OR “Iraq” OR “Jamaica” OR “Jordan” OR “Kazakhstan” OR “Kenya” OR “Kiribati” OR “Kyrgyzstan” OR “Laos” OR “Lebanon” OR “Lesotho” OR “Liberia” OR “Libya” OR “Madagascar” OR “Malawi” OR “Malaysia” OR “Maldives” OR “Mali” OR “Mauritania” OR “Mauritius” OR “Morocco” OR “Mozambique” OR “Myanmar” OR “Namibia” OR “Nicaragua” OR “Niger” OR “Nigeria” OR “Palau” OR “Panama” OR “Benin” OR “Peru” OR “Philippines” OR “Georgia” OR “Macedonia” OR “Rwanda” OR “Saint Kitts” OR “Saint Vincent” OR “Saint Lucia” OR “Sao Tome Principe” OR “Senegal” OR “Serbia” OR “Seychelles” OR “Sierra Leone” OR “South Africa” OR “Solomon Islands” OR “Somalia” OR “Sudan” OR “Swaziland” OR “Tajikistan” OR “Tanzania” OR “Thailand” OR “Togo” OR “Tonga” OR “Trinidad” OR “Tobago” OR “Tunisia” OR “Turkmenistan” OR “Uganda” OR “Ukraine” OR “Uzbekistan” OR “Vanuatu” OR “Vietnam” OR “Samoa” OR “Zambia” OR “Zimbabwe”) or AB (“Russia” or “China” or “Brazil” or “Brasil” or “Bosnia” or “Herzegovina” or “Kuwait” or “Moldova” or “Mongolia” or “Montenegro” or “Nepal” or “Oman” or “Pakistan” or “Paraguay” or “Puerto Rico” or “Kosovo” or “Yemen” or “Romania” or “Saudi Arabia” or “Sri Lanka” or “Suriname” or “Syria” or “Uruguay” or “Venezuela” or “Qatar” or “United Arab“ or “United Arab Emirates” or “Colombia” or “Afghanistan” OR “Africa” OR “Albania” OR “Algeria” OR “Angola” OR “Antigua” OR “Argentina” OR “Armenia” OR “Azerbaijan” OR “Bangladesh” OR “Barbados” OR “Barbuda” OR “Bahrain” OR “Belarus” OR “Belize” OR “Bhutan” OR “Bolivia” OR “Botswana” OR “Bulgaria” OR “Burkina Faso” OR “Burundi” OR “Cambodia” OR “Cameroon” OR “Chad” OR “Comoros” OR “Congo” OR “Costa Rica” OR “Croatia” OR “Cuba” OR “Congo” OR “Djibouti” OR “Dominica” OR “Dominican” OR “East Timor” OR “Ecuador” OR “Egypt” OR “El Salvador” OR “Guinea” OR “Eritrea” OR “Ethiopia” OR “Fiji” OR “Gabon” OR “Gambia” OR “Ghana” OR “Grenada” OR “Guatemala” OR “Guinea” OR “Guyana” OR “Haiti” OR “Honduras” OR “India” OR “Indonesia” OR “Iran” OR “Iraq” OR “Jamaica” OR “Jordan” OR “Kazakhstan” OR “Kenya” OR “Kiribati” OR “Kyrgyzstan” OR “Laos” OR “Lebanon” OR “Lesotho” OR “Liberia” OR “Libya” OR “Madagascar” OR “Malawi” OR “Malaysia” OR “Maldives” OR “Mali” OR “Mauritania” OR “Mauritius” OR “Morocco” OR “Mozambique” OR “Myanmar” OR “Namibia” OR “Nicaragua” OR “Niger” OR “Nigeria” OR “Palau” OR “Panama” OR “Benin” OR “Peru” OR “Philippines” OR “Georgia” OR “Macedonia” OR “Rwanda” OR “Saint Kitts” OR “Saint Vincent” OR “Saint Lucia” OR “Sao Tome Principe” OR “Senegal” OR “Serbia” OR “Seychelles” OR “Sierra Leone” OR “South Africa” OR “Solomon Islands” OR “Somalia” OR “Sudan” OR “Swaziland” OR “Tajikistan” OR “Tanzania” OR “Thailand” OR “Togo” OR “Tonga” OR “Trinidad” OR “Tobago” OR “Tunisia” OR “Turkmenistan” OR “Uganda” OR “Ukraine” OR “Uzbekistan” OR “Vanuatu” OR “Vietnam” OR “Samoa” OR “Zambia” OR “Zimbabwe”)

S19 "sensory aid*" or "hearing aid*" or "decision aid*" or "smoking cessation aid*"

S18 "cost analys*" or "cost benefit" or "cost effectiv*" or "cost minimi*" or "cost utility" or "health cost*" or "healthcare cost*" or "direct cost*" or "indirect cost*" or "health care cost*" or "health expenditure*" or "burden of illness*" or "burden of disease*" or "economic burden" or "economic evaluation*" or "health* economic*" or "cost* of illness*" or "illness* cost*" or "cost* of disease*" or "disease* cost*" or "sickness cost*" or "productivity cost*" or "cost* and benefit*"

S17 TI ("Hepatitis B") or AB ("Hepatitis B")

S16 TI ("Genital wart*" or "venereal wart*" or "condylomata acuminata") or AB ("Genital wart*" or "venereal wart*" or "condylomata acuminata")

S15 TI ("HPV infection*" or "Human Papillomavirus Infection*") or AB ("HPV infection*" or "Human Papillomavirus Infection*")

S14 TI ("Pelvic Inflammatory Disease*") or AB ("Pelvic Inflammatory Disease*")

S13 TI ("trichomonas") or AB ("trichomonas")

S12 TI ("Gonorrh*") or AB ("Gonorrh*")

S11 TI ("Chlamydia trachomatis") or AB ("Chlamydia trachomatis")

S10 TI ("Syphilis") or AB ("Syphilis")

S9 TI ("genital herpes" or "Herpes Genitalis" or "genital infection") or AB ("genital herpes" or "Herpes Genitalis" or "genital infection")

S8 TI ("HIV" or "human immun* deficiency virus" or "human immunodeficiency virus") or AB ("HIV" or "human immun* deficiency virus" or "human immunodeficiency virus")

S7 TI ("AIDS" or "acquired immun* deficiency syndrome" or "acquired immunodeficiency syndrome") or AB ("AIDS" or "acquired immun* deficiency syndrome" or "acquired immunodeficiency syndrome")

S6 TI ( "STI" or "STIs" or "STD" or "STDs" OR “venereal disease” OR “venereal diseases” ) OR AB ( "STI" or "STIs" or "STD" or "STDs" OR “venereal disease” OR “venereal diseases” )

S5 "Sexually Transmitted Disease*" or "Sexually Transmitted infection*"

S4 "contraception*" or "contraceptions" or "contraceptive" or "contraceptives" or "birth control"

S3 "sexual behavior*" or "sexual behaviour*"

S2 "sex* education"

S1 "sexual health"

# NHS EED

[27 May 2019]

1 (sexual health) IN NHSEED

2 (sex* behavi*) IN NHSEED

3 (safe sex) IN NHSEED

4 (unsafe sex) IN NHSEED

5 MeSH DESCRIPTOR Sex Education EXPLODE ALL TREES

6 (STI or STIs or STD or STDs) IN NHSEED

7 MeSH DESCRIPTOR Syphilis EXPLODE ALL TREES

8 MeSH DESCRIPTOR Syphilis, Congenital EXPLODE ALL TREES

9 MeSH DESCRIPTOR Chlamydia trachomatis EXPLODE ALL TREES

10 MeSH DESCRIPTOR Neisseria gonorrhoeae EXPLODE ALL TREES

11 MeSH DESCRIPTOR Gonorrhea EXPLODE ALL TREES

12 MeSH DESCRIPTOR Trichomonas Infections EXPLODE ALL TREES

13 MeSH DESCRIPTOR Trichomonas vaginalis EXPLODE ALL TREES

14 MeSH DESCRIPTOR Pelvic Inflammatory Disease EXPLODE ALL TREES

15 MeSH DESCRIPTOR Papillomaviridae EXPLODE ALL TREES

16 MeSH DESCRIPTOR Hepatitis B EXPLODE ALL TREES

17 MeSH DESCRIPTOR Hepatitis B virus EXPLODE ALL TREES

18 (contraception):TI OR (contraceptions):TI IN NHSEED

19 (contraceptive):TI OR (contraceptives):TI IN NHSEED

20 (HIV):TI OR (Human immunodeficiency virus):TI OR (Human immun* deficiency virus):TI IN NHSEED

21 (AIDS):TI OR (acquired immun* deficiency syndrome):TI OR (acquired immunodeficiency syndrome):TI IN NHSEED

22 (herpes genitalis):TI OR (genital herpes):TI OR (genital infection):TI IN NHSEED

23 (syphilis):TI IN NHSEED

24 (chlamydia trachomatis):TI IN NHSEED

25 (Gonorrh*):TI IN NHSEED

26 (Trichomonas vaginali*):TI OR (Trichomonas infection*):TI IN NHSEED

27 (Pelvic inflammatory infection):TI OR (Pelvic inflammatory infections):TI IN NHSEED

28 (Human papillomavirus):TI OR (Human Papilloma Virus):TI IN NHSEED

29 (Genital wart):TI OR (Condylomata Acuminata):TI OR (Genital warts):TI IN NHSEED

30 MeSH DESCRIPTOR Condylomata Acuminata EXPLODE ALL TREES

31 (hepatitis B):TI IN NHSEED

32 (MeSH DESCRIPTOR Sex Education EXPLODE ALL TREES) IN NHSEED

33 (MeSH DESCRIPTOR Sexually Transmitted Diseases EXPLODE ALL TREES) IN NHSEED

34 (MeSH DESCRIPTOR Contraception Behavior EXPLODE ALL TREES) IN NHSEED

35 (MeSH DESCRIPTOR Contraception EXPLODE ALL TREES) IN NHSEED

36 (MeSH DESCRIPTOR HIV EXPLODE ALL TREES) IN NHSEED

37 (MeSH DESCRIPTOR Acquired Immunodeficiency Syndrome EXPLODE ALL TREES) IN NHSEED

38 (MeSH DESCRIPTOR Herpes Genitalis EXPLODE ALL TREES) IN NHSEED

39 (MeSH DESCRIPTOR Syphilis EXPLODE ALL TREES) IN NHSEED

40 (MeSH DESCRIPTOR Syphilis, Congenital EXPLODE ALL TREES) IN NHSEED

41 (MeSH DESCRIPTOR Chlamydia trachomatis EXPLODE ALL TREES) IN NHSEED

42 (MeSH DESCRIPTOR Neisseria gonorrhoeae EXPLODE ALL TREES) IN NHSEED

43 (MeSH DESCRIPTOR Gonorrhea EXPLODE ALL TREES) IN NHSEED

44 (MeSH DESCRIPTOR Trichomonas Infections EXPLODE ALL TREES) IN NHSEED

45 (MeSH DESCRIPTOR Trichomonas vaginalis EXPLODE ALL TREES) IN NHSEED

46 (MeSH DESCRIPTOR Pelvic Inflammatory Disease EXPLODE ALL TREES) IN NHSEED

47 (MeSH DESCRIPTOR Papillomaviridae EXPLODE ALL TREES) IN NHSEED

48 (MeSH DESCRIPTOR Hepatitis B EXPLODE ALL TREES) IN NHSEED

49 (MeSH DESCRIPTOR Hepatitis B virus EXPLODE ALL TREES) IN NHSEED

50 (MeSH DESCRIPTOR Condylomata Acuminata EXPLODE ALL TREES) IN NHSEED FROM 1999 TO 2019

51 #1 OR #2 OR #3 OR #4 OR #6 OR #18 OR #19 OR #20 OR #21 OR #22 OR #23 OR #24 OR #25 OR #26 OR #27 OR #28 OR #29 OR #31 OR #32 OR #33 OR #34 OR #35 OR #36 OR #37 OR #38 OR #39 OR #40 OR #41 OR #42 OR #43 OR #44 OR #45 OR #46 OR #47 OR #48 OR #49 OR #50

52 (sensory aid*) OR (hearing aid*) OR (decision aid*) IN NHSEED

53 (animal) OR (animals) OR (nonhuman) IN NHSEED

54 (#1 OR #2 OR #3 OR #4 OR #6 OR #18 OR #19 OR #20 OR #21 OR #22 OR #23 OR #24 OR #25 OR #26 OR #27 OR #28 OR #29 OR #31 OR #32 OR #33 OR #34 OR #35 OR #36 OR #37 OR #38 OR #39 OR #40 OR #41 OR #42 OR #43 OR #44 OR #45 OR #46 OR #47 OR #48 OR #49 OR #50) IN NHSEED FROM 1999 TO 2019

55 (#1 OR #2 OR #3 OR #4 OR #6 OR #18 OR #19 OR #20 OR #21 OR #22 OR #23 OR #24 OR #25 OR #26 OR #27 OR #28 OR #29 OR #31 OR #32 OR #33 OR #34 OR #35 OR #36 OR #37 OR #38 OR #39 OR #40 OR #41 OR #42 OR #43 OR #44 OR #45 OR #46 OR #47 OR #48 OR #49 OR #50) FROM 1999 TO 2019

# PUBMED

*The original search was updated to also include studies between 2019 and June 2021 (time of update). Studies were only searched in PubMed.*

Search: ((((("Sexual Behavior"[MeSH] OR sexual behavior*[tiab] OR sexual behaviour*[tiab] OR sexual activit*[tiab] OR sexual education[tiab] OR sex education[tiab] OR sexuality education[tiab] OR sexual health[tiab] OR "Sexual Health"[MeSH] OR safe sex[tiab] OR safe* sex[tiab] OR "Unsafe Sex"[MeSH] OR "Contraception"[MeSH] OR contracepti*[tiab] OR birth control[tiab] OR ("Sexually Transmitted Diseases"[MeSH] OR sexually transmitted disease*[tiab] OR sexually transmitted infection*[tiab] OR STD[tiab] OR STDs[tiab] OR STI[tiab] OR STIs[tiab] OR venereal disease*[tiab] OR "Acquired Immunodeficiency Syndrome"[MeSH] OR acquired immune deficiency syndrome*[tiab] OR Acquired Immunodeficiency Syndrome*[tiab] OR acquired immuno deficiency syndrome*[tiab] OR Acquired Immunologic deficiency Syndrome*[tiab] OR AIDS[tiab] OR "HIV"[MeSH] OR HIV[tiab] OR human immunodeficiency virus*[tiab] OR herpes genitalis[tiab] OR genital herpes[tiab] OR "Syphilis"[MeSH] OR syphilis[tiab] OR "Chlamydia"[MeSH] OR chlamydia[tiab] OR "Gonorrhea"[MeSH] OR gonorrh*[tiab] OR "Trichomonas Infections"[MeSH] OR trichomonas infection*[tiab] OR trichomonias*[tiab] OR "Pelvic Inflammatory Disease"[MeSH] OR pelvic inflammatory disease*[tiab] OR inflammatory pelvic disease*[tiab] OR HPV[tiab] OR human papillomavirus*[tiab] OR condylomata acuminata[tiab] OR genital wart*[tiab] OR venereal wart*[tiab] OR "Hepatitis B"[MeSH] OR "Hepatitis B"[tiab])))) AND ((cost*[tiab] OR "costs and cost analysis"[MeSH:noexp] OR cost benefit analys*[Tiab] OR "cost-benefit analysis"[MeSH] OR "health care costs"[MeSH:noexp] OR "Cost of Illness"[MeSH] OR health expenditure[tiab] OR health expenditure*[tiab]))) NOT (((Afghanistan[Title] OR Africa[Title] OR Albania[Title] OR Algeria[Title] OR Angola[Title] OR Antigua[Title] OR Argentina[Title] OR Armenia[Title] OR Azerbaijan[Title] OR Bangladesh[Title] OR Barbados[Title] OR Barbuda[Title] OR Belarus[Title] OR Belize[Title] OR Brazil[Title] OR Bhutan[Title] OR Bolivia[Title] OR Bosnia[Title] OR Botswana[Title] OR Bulgaria[Title] OR Burkina Faso[Title] OR Burundi[Title] OR Cambodia[Title] OR Cameroon[Title] OR Central African Republic[Title] OR Chad[Title] OR Colombia[Title] OR Comoros[Title] OR Congo[Title] OR Costa Rica[Title] OR Croatia[Title] OR Cuba[Title] OR Czech*[Title] OR Congo[Title] OR Djibouti[Title] OR Dominica[Title] OR Dominican[Title] OR East Timor[Title] OR Ecuador[Title] OR Egypt[Title] OR El Salvador[Title] OR Equatorial Guinea[Title] OR Eritrea[Title] OR Estonia[Title] OR Ethiopia[Title] OR Fiji[Title] OR Gabon[Title] OR Gambia[Title] OR Ghana[Title] OR Grenada[Title] OR Guatemala[Title] OR Guinea-Bissau[Title] OR Guyana[Title] OR Haiti[Title] OR Honduras[Title] OR Hungary[Title] OR India[Title] OR Indonesia[Title] OR Iran[Title] OR Iraq[Title] OR Ivory Coast[Title] OR Jamaica[Title] OR Jordan[Title] OR Kazakhstan[Title] OR Kenya[Title] OR Kiribati[Title] OR Kyrgyzstan[Title] OR Laos[Title] OR Latvia[Title] OR Lebanon[Title] OR Lesotho[Title] OR Liberia[Title] OR Libya[Title] OR Lithuania[Title] OR Madagascar[Title] OR Malawi[Title] OR Malaysia[Title] OR Maldives[Title] OR Mali[Title] OR Marshall Islands[Title] OR Mauritania[Title] OR Mauritius[Title] OR Micronesia[Title] OR Moldova[Title] OR Mongolia[Title] OR Montenegro[Title] OR Morocco[Title] OR Mozambique[Title] OR Myanmar[Title] OR Namibia[Title] OR Nepal[Title] OR New Guinea[Title] OR Nicaragua[Title] OR Niger[Title] OR Nigeria[Title] OR Oman[Title] OR Pakistan[Title] OR Palau[Title] OR Panama[Title] OR Papua New Guinea[Title] OR Paraguay[Title] OR Benin[Title] OR China[Title] OR Peru[Title] OR Philippines[Title] OR Poland[Title] OR Cape Verde[Title] OR Georgia[Title] OR Kosovo[Title] OR Macedonia[Title] OR Yemen[Title] OR Romania[Title] OR Russia[Title] OR Rwanda[Title] OR Saint Kitts[Title] OR Saint Vincent[Title] OR Saint Lucia[Title] OR Sao Tome Principe[Title] OR Saudi Arabia[Title] OR Senegal[Title] OR Serbia[Title] OR Seychelles[Title] OR Sierra Leone[Title] OR Slovak*[Title] OR South Africa[Title] OR Solomon Islands[Title] OR Somalia[Title] OR Sri Lanka[Title] OR Sri-Lanka[Title] OR Sudan[Title] OR Suriname[Title] OR Swaziland[Title] OR Syria[Title] OR Tajikistan[Title] OR Tanzania[Title] OR Thailand[Title] OR Togo[Title] OR Tonga[Title] OR Trinidad[Title] OR Tobago[Title] OR Tunisia[Title] OR Turkey[Title] OR Turkmenistan[Title] OR Uganda[Title] OR Ukraine[Title] OR Uruguay[Title] OR Uzbekistan[Title] OR Vanuatu[Title] OR Venezuela[Title] OR Vietnam[Title] OR Samoa[Title] OR Zambia[Title] OR Zimbabwe[Title])))) NOT ((developing countr*[Title] OR underdeveloped countr*[Title] OR under developed countr*[Title] OR "Latin America"[Mesh] OR "Africa"[MeSH] OR "Asia, Northern"[ MeSH] OR "Asia, Central"[MeSH] OR "Asia, Southeastern"[MeSH] OR "Asia, Western"[MeSH] OR "Central America"[MeSH] OR "Caribbean Region"[MeSH] OR Latin America[Title] OR Africa[Title] OR Central America[Title] OR Caribbean Region[Title])) Filters: Humans, from 2019/5/25 - 2021/6/30
