# Supplementary material for: Are intersectoral costs considered in economic evaluations of interventions relating to sexually transmitted infections (STIs)? A systematic review
Source: BMC Public Health. 2022 Nov 25;22:2180. doi: 10.1186/s12889-022-14484-z (PMC9701033; doi:10.1186/s12889-022-14484-z)
Supplement: Supplementary file 3 — Additional file 3. [file 12889_2022_14484_MOESM3_ESM.docx]

Supplementary file 3: PICO table

**Participants/population**

This review focuses on full (trial-based or model-based) economic evaluations of interventions including individuals (10 years and older) living in Organisation for Economic Co-operation and Development (OECD) member countries and at risk of contracting STIs and individuals infected with an STI

**Intervention(s), exposure(s)**

This review considers different types of interventions implemented in OECD member countries and aimed at preventing, controlling or treating STIs or promoting healthy (sexual) behaviour

**Comparator(s)/control**

Any comparator including usual care, treatment as usual or no intervention are considered

**Main outcome(s)**

Any cost and outcome relevant to a full economic evaluation

**Study design**

Trial-based or model-based economic evaluations that adopted a societal perspective
