# Supplementary material for: Are intersectoral costs considered in economic evaluations of interventions relating to sexually transmitted infections (STIs)? A systematic review
Source: BMC Public Health. 2022 Nov 25;22:2180. doi: 10.1186/s12889-022-14484-z (PMC9701033; doi:10.1186/s12889-022-14484-z)
Supplement: Supplementary file 4 — Additional file 4. [file 12889_2022_14484_MOESM4_ESM.docx]

Supplemental file 4: Studies excluded at full-text

| Brisson, M., Laprise, J. F., Chesson, H. W., Drolet, M., Malagón, T., Boily, M. C., & Markowitz, L. E. (2016). Health and economic impact of switching from a 4-valent to a 9-valent HPV vaccination program in the United States. *JNCI: Journal of the National Cancer Institute*, *108*(1). |
| --- |
| Colchero, M. A., Bautista-Arredondo, S., Cortés-Ortiz, M. A., Romero-Martinez, M., Salas, J., Sosa-Rubí, S. G., & Uribe, P. (2016). Impact and economic evaluations of a combination prevention programme for men who have sex with men in Mexico. *Aids*, *30*(2), 293-300. |
| Dowdy, D. W., Rodriguez, R. M., Bradley Hare, C., & Kaplan, B. (2011). Cost‐effectiveness of targeted human immunodeficiency virus screening in an urban emergency department. *Academic Emergency Medicine*, *18*(7), 745-753. |
| Farnham, P. G., Sansom, S. L., & Hutchinson, A. B. (2012). How much should we pay for a new HIV diagnosis? A mathematical model of HIV screening in US clinical settings. *Medical Decision Making*, *32*(3), 459-469. |
| Leibowitz, A. A., Harawa, N., Sylla, M., Hallstrom, C. C., & Kerndt, P. R. (2013). Condom distribution in jail to prevent HIV infection. *AIDS and Behavior*, *17*(8), 2695-2702. |
| Holtgrave, D. R., Maulsby, C., Kharfen, M., Jia, Y., Wu, C., Opoku, J., ... & Pappas, G. (2012). Cost–utility analysis of a female condom promotion program in Washington, DC. *AIDS and Behavior*, *16*(5), 1115-1120. |
| Hutchinson, A. B., Patel, P., Sansom, S. L., Farnham, P. G., Sullivan, T. J., Bennett, B., ... & Branson, B. M. (2010). Cost-effectiveness of pooled nucleic acid amplification testing for acute HIV infection after third-generation HIV antibody screening and rapid testing in the United States: a comparison of three public health settings. *PLoS medicine*, *7*(9), e1000342. |
| Ladapo, J. A., Elliott, M. N., Bogart, L. M., Kanouse, D. E., Vestal, K. D., Klein, D. J., ... & Schuster, M. A. (2013). Cost of talking parents, healthy teens: A worksite-based intervention to promote parent–adolescent sexual health communication. *Journal of Adolescent Health*, *53*(5), 595-601. |
| Long, E. F., Brandeau, M. L., & Owens, D. K. (2010). The cost-effectiveness and population outcomes of expanded HIV screening and antiretroviral treatment in the United States. *Annals of internal medicine*, *153*(12), 778-789. |
| Stevens, E. R., Nucifora, K., Zhou, Q., Braithwaite, R. S., Cleland, C. M., Ritchie, A. S., ... & Gwadz, M. V. (2018). Cost-effectiveness of peer-versus venue-based approaches for detecting undiagnosed HIV among heterosexuals in high-risk new York City neighborhoods. *Journal of acquired immune deficiency syndromes (1999)*, *77*(2), 183. |
| Suijkerbuijk, A., Over, E., Aar, F., Götz, H., Benthem, B., & Lugnér, A. (2017). VP35 Economic Consequences Of A Restricted Dutch Sexually Transmitted Infection-Testing Policy. *International Journal of Technology Assessment in Health Care*, *33*(S1), 165-165. |
| Tuite, A. R., Jayaraman, G. C., Allen, V. G., & Fisman, D. N. (2012). Estimation of the burden of disease and costs of genital Chlamydia trachomatis infection in Canada. *Sexually transmitted diseases*, 260-267. |
| Juusola, J. L., Brandeau, M. L., Owens, D. K., & Bendavid, E. (2012). The cost-effectiveness of preexposure prophylaxis for HIV prevention in the United States in men who have sex with men. *Annals of internal medicine*, *156*(8), 541-550. |
| Spaulding, A. C., Pinkerton, S. D., Superak, H., Cunningham, M. J., Resch, S., Jordan, A. O., & Yang, Z. (2013). Cost analysis of enhancing linkages to HIV care following jail: a cost-effective intervention. *AIDS and Behavior*, *17*(2), 220-226. |
| Hersh, A. R., Megli, C. J., & Caughey, A. B. (2018). Repeat screening for syphilis in the third trimester of pregnancy: a cost-effectiveness analysis. *Obstetrics & Gynecology*, *132*(3), 699-707. |
| Wang, L. Y., Hamilton, D. T., Rosenberg, E. S., Aslam, M. V., Sullivan, P. S., Katz, D. A., ... & Goodreau, S. M. (2020). Cost-effectiveness of pre-exposure prophylaxis among adolescent sexual minority males. *Journal of Adolescent Health*, *66*(1), 100-106. |
| Kazemian, P., Costantini, S., Kumarasamy, N., Paltiel, A. D., Mayer, K. H., Chandhiok, N., ... & Freedberg, K. A. (2020). The Cost-effectiveness of Human Immunodeficiency Virus (HIV) Preexposure Prophylaxis and HIV Testing Strategies in High-risk Groups in India. *Clinical Infectious Diseases*, *70*(4), 633-642. |
| Bogaards, J. A., Coupé, V. M., Meijer, C. J., & Berkhof, J. (2011). The clinical benefit and cost-effectiveness of human papillomavirus vaccination for adult women in the Netherlands. *Vaccine*, *29*(48), 8929-8936. |
| Anderson, J., Wilson, D., Templeton, D. J., Grulich, A., Carter, R., & Kaldor, J. (2009). Cost-effectiveness of adult circumcision in a resource-rich setting for HIV prevention among men who have sex with men. *The Journal of infectious diseases*, *200*(12), 1803-1812. |
