# Supplementary material for: Are intersectoral costs considered in economic evaluations of interventions relating to sexually transmitted infections (STIs)? A systematic review
Source: BMC Public Health. 2022 Nov 25;22:2180. doi: 10.1186/s12889-022-14484-z (PMC9701033; doi:10.1186/s12889-022-14484-z)
Supplement: Supplementary file 5 — Additional file 5. [file 12889_2022_14484_MOESM5_ESM.docx]

Supplemental file 5: Intersectoral cost components included per individual study

|  | Patient & Family | | | | Informal Care | | | Paid Labour (productivity) | | | | | | | | Non-paid opportunity costs (productivity) | Education | Consumption |
| --- | --- | --- | --- | --- | --- | --- | --- | --- | --- | --- | --- | --- | --- | --- | --- | --- | --- | --- |
|  | Patient time (and travel) | Travel expenses | Out-of-pocket costs | Premature burial costs | Informal caregiver support (non-family) | Care provided by family/friends | Productivity loss due to absenteeism | | Productivity loss due to presenteeism | Lost income | Lost revenue due to unemployment rate gap | Fringe benefits | Early retirement | Avoided future production loss | Intervention-related productivity gains (cost savings) | Inability to perform unpaid work/activities i.e. domestic tasks or voluntary work | School absence | Future consumption unrelated to health |
| Adamson  et al. |  |  | X |  |  |  | X | |  | X |  | X |  |  |  | X |  | X |
| Campos  et al. | X | X |  |  |  |  |  | |  |  |  |  |  |  |  |  |  |  |
| Coupe  et al. |  | X |  |  |  |  | X | |  |  |  |  |  |  |  |  |  |  |
| Damm  et al. |  |  |  |  |  |  | X | |  |  |  |  |  |  |  |  |  |  |
| De Kok  et al. | X |  |  |  |  |  |  | |  |  |  |  |  |  |  |  |  |  |
| De Wit  et al. |  |  |  |  |  |  | X | |  |  |  |  |  |  |  |  |  |  |
| Deogan  et al. |  |  |  |  |  |  | X | |  |  |  |  |  | X |  |  |  |  |
| Drabo  et al. |  |  |  |  | X | X | X | |  |  |  |  |  |  |  |  |  |  |
| Fogelberg  et al. | X |  |  |  |  |  |  | |  |  |  |  |  |  |  |  |  |  |
| Gift  et al. |  | X |  |  |  |  | X | |  |  |  |  |  |  |  |  |  |  |
| Ginsberg  et al. |  | X |  | X |  |  | X | |  |  |  |  |  |  |  |  |  |  |
| Kim  et al. | X | X |  |  |  |  |  | |  |  |  |  |  |  |  |  |  |  |
| Kim &  Goldie | X | X |  |  |  |  |  | |  |  |  |  |  |  |  |  |  |  |
| Krauth  et al. |  |  |  |  |  |  | X | | X |  |  |  | X |  |  |  |  |  |
| Mahumud  et al. |  |  |  |  |  | X | X | |  |  |  |  |  |  |  | X |  |  |
| Nosyk  et al. |  |  |  |  |  |  |  | |  |  |  |  |  |  | X |  |  |  |
| Ouellet  et al. |  |  |  |  |  |  | X | |  |  | X |  |  |  |  |  |  |  |
| Owusu-Edusei  et al. (2015) |  |  |  |  |  |  | X | |  |  |  |  |  |  |  |  |  |  |
| Owusu-Edusei  et al. (2016) |  |  |  |  |  |  | X | |  |  |  |  |  |  |  |  |  |  |
| Regnier  et al. |  |  |  |  |  |  |  | |  | X |  |  |  |  | X |  |  |  |
| Rogoza  et al. |  | X |  |  |  |  | X | |  |  |  |  |  |  |  |  |  |  |
| Rossi  et al. |  |  | X |  |  | X | X | |  |  |  |  |  |  |  |  |  |  |
| Rours  et al. |  |  |  |  |  |  | X | |  |  |  |  |  |  |  |  |  |  |
| Van Luenen  et al. |  |  |  |  |  |  | X | | X |  |  |  |  |  |  | X |  |  |
| Van Wifferen  et al. |  | X |  |  |  |  | X | |  |  |  |  |  |  |  |  |  |  |
| Wijnen  et al. |  |  |  |  |  | X | X | |  |  |  |  |  |  |  | X | X |  |
| Wolff  et al. |  |  |  |  |  |  | X | |  |  |  |  |  |  |  |  |  |  |
| Zechmeister  et al. |  |  |  |  |  |  | X | |  |  |  |  |  |  |  |  |  |  |
| Zulliger  et al. |  | X |  |  |  |  | X | |  |  |  |  |  |  |  |  |  |  |
